# Pharmacodynamic evaluation and safety assessment of treatment with antibodies to serum amyloid P component in patients with cardiac amyloidosis: an open-label Phase 2 study and an adjunctive immuno-PET imaging study

**DOI:** 10.1186/s12872-021-02407-6

**Published:** 2022-02-13

**Authors:** Ashutosh Wechalekar, Gunnar Antoni, Wasfi Al Azzam, Mats Bergström, Swethajit Biswas, Chao Chen, Joseph Cheriyan, Matthew Cleveland, Louise Cookson, Paul Galette, Robert L. Janiczek, Raymond Y. Kwong, Mary Ann Lukas, Helen Millns, Duncan Richards, Ian Schneider, Scott D. Solomon, Jens Sörensen, James Storey, Douglas Thompson, Guus van Dongen, Danielle J. Vugts, Anders Wall, Gerhard Wikström, Rodney H. Falk

**Affiliations:** 1 University College London, Gower Street, Bloomsbury, London, WC1E 6BT UK; 2 Institutionen för Medicinska Vetenskaper, Uppsala University, Uppsala, Sweden; 3 GlaxoSmithKline, Philadelphia, USA; 4 GlaxoSmithKline, Stevenage, Hertfordshire UK; 5 Cambridge University Hospitals NHS Foundation Trust, Cambridge, UK; 6 GlaxoSmithKline, Cambridge, UK; 7 Brigham and Women’s Hospital, Boston, MA USA; 8 Amsterdam UMC, VU University, Amsterdam, The Netherlands; 9 Present Address: Takeda, Lexington, MA USA; 10 Present Address: Nuffield Department of Orthopaedics, Rheumatology and Musculoskeletal Sciences, University of Oxford, Oxford, UK; 11 Present Address: Consolidated Consulting LTD, Cambridge, UK

**Keywords:** Cardiac amyloidosis, Miridesap, Dezamizumab, Positron emission tomography, Immuno-PET, Serum amyloid P component, Systemic amyloidosis

## Abstract

**Background:**

In a Phase I study treatment with the serum amyloid P component (SAP) depleter miridesap followed by monoclonal antibody to SAP (dezamizumab) showed removal of amyloid from liver, spleen and kidney in patients with systemic amyloidosis. We report results from a Phase 2 study and concurrent immuno-positron emission tomography (PET) study assessing efficacy, pharmacodynamics, pharmacokinetics, safety and cardiac uptake (of dezamizumab) following the same intervention in patients with cardiac amyloidosis.

**Methods:**

Both were uncontrolled open-label studies. After SAP depletion with miridesap, patients received ≤ 6 monthly doses of dezamizumab in the Phase 2 trial (n = 7), ≤ 2 doses of non-radiolabelled dezamizumab plus [^89^Zr]Zr-dezamizumab (total mass dose of 80 mg at session 1 and 500 mg at session 2) in the immuno-PET study (n = 2). Primary endpoints of the Phase 2 study were changed from baseline to follow-up (at 8 weeks) in left ventricular mass (LVM) by cardiac magnetic resonance imaging and safety. Primary endpoint of the immuno-PET study was [^89^Zr]Zr-dezamizumab cardiac uptake assessed via PET.

**Results:**

Dezamizumab produced no appreciable or consistent reduction in LVM nor improvement in cardiac function in the Phase 2 study. In the immuno-PET study, measurable cardiac uptake of [^89^Zr]Zr-dezamizumab, although seen in both patients, was moderate to low. Uptake was notably lower in the patient with higher LVM. Treatment-associated rash with cutaneous small-vessel vasculitis was observed in both studies. Abdominal large-vessel vasculitis after initial dezamizumab dosing (300 mg) occurred in the first patient with immunoglobulin light chain amyloidosis enrolled in the Phase 2 study. Symptom resolution was nearly complete within 24 h of intravenous methylprednisolone and dezamizumab discontinuation; abdominal computed tomography imaging showed vasculitis resolution by 8 weeks.

**Conclusions:**

Unlike previous observations of visceral amyloid reduction, there was no appreciable evidence of amyloid removal in patients with cardiac amyloidosis in this Phase 2 trial, potentially related to limited cardiac uptake of dezamizumab as demonstrated in the immuno-PET study. The benefit-risk assessment for dezamizumab in cardiac amyloidosis was considered unfavourable after the incidence of large-vessel vasculitis and development for this indication was terminated.

*Trial registration* NCT03044353 (2 February 2017) and NCT03417830 (25 January 2018).

**Supplementary Information:**

The online version contains supplementary material available at 10.1186/s12872-021-02407-6.

## Background

Systemic amyloidosis is a progressive, usually fatal disorder in which misfolded proteins form fibrils that accumulate in vital organs leading to progressive organ dysfunction [1, 2]. The two most common types are immunoglobulin light chain (AL) and transthyretin (ATTR) amyloidosis [2]; cardiac involvement is common in both forms [1]. Prognosis is poor for both types, but significantly worse for patients with cardiac AL, with an overall median survival of approximately 9 months, or 3.5 months for patients with markedly elevated N-terminal pro-B-type natriuretic peptide (NT-proBNP) and cardiac troponin [3, 4].

Current treatments for cardiac amyloidosis target the production of precursor proteins by chemotherapy for AL [1, 5], and oligonucleotide gene silencing therapies [6] or targetted protein stabilisers (tafamidis) [7] for ATTR. However, no approved therapies directly remove cardiac amyloid deposits, which can range from mild to extensive on diagnosis [1, 5, 8].

In a Phase 1 clinical trial we demonstrated progressive removal of amyloid with up to three cycles of a therapy that targetted the plasma protein serum amyloid P component (SAP), which is universally present in amyloid deposits, and is therefore a target in all forms of amyloidosis [9]. For each session of treatment, normal circulating SAP was first acutely depleted by miridesap [10, 11], leaving residual SAP within amyloid deposits. This was then targetted by the anti-SAP monoclonal antibody (mAb) dezamizumab, which in mice, has been shown to lead to removal of amyloid through a macrophage giant cell response [11, 12]. Depletion of amyloid in humans was initially demonstrated in several organs (liver, spleen and kidney) in different forms of amyloidosis (AL, AA [amyloid A protein], fibrinogen and apolipoprotein A-I) [13, 14]. Based on these encouraging Phase 1 results, we designed a Phase 2 study that aimed to investigate the impact of treatment on cardiac amyloid deposits, as well as the occurrence and nature of dermatological adverse events (AEs; mainly rashes), which were seen in 58% of patients in the Phase 1 study and seemed to occur with incremental dezamizumab doses. The Phase 2 study concentrated on patients with either AL or ATTR cardiomyopathy (ATTR-CM), and subjects were intended to receive up to 6 treatments of miridesap followed by dezamizumab (anti-SAP treatment) at monthly intervals. Successful development of [^89^Zr]Zr-DFO-NCS-dezamizumab ([^89^Zr]Zr-dezamizumab) enabled a concurrent immuno-positron emission tomography (PET) study to be performed, which specifically assessed cardiac uptake and biodistribution of [^89^Zr]Zr-dezamizumab.

We report data from the Phase 2 trial in concert with the Phase 1 PET imaging study, to demonstrate the interplay of the data from both studies in informing a benefit-risk assessment that led to the termination of development of this anti-SAP treatment for systemic amyloidosis.

## Methods

### Phase 2 study

#### Study design and treatment

This was an open-label, non-randomised study in patients with systemic amyloidosis and cardiac dysfunction caused by cardiac amyloidosis (NCT03044353). Three groups were planned: patients with ATTR-CM (Group 1), post-chemotherapy AL amyloidosis (Group 2) and newly diagnosed Mayo stage II or IIIa AL amyloidosis (Group 3). Group 1 and Group 2 began recruitment in parallel; no patients were recruited into Group 3 due to early study termination.

**Fig. 1:**
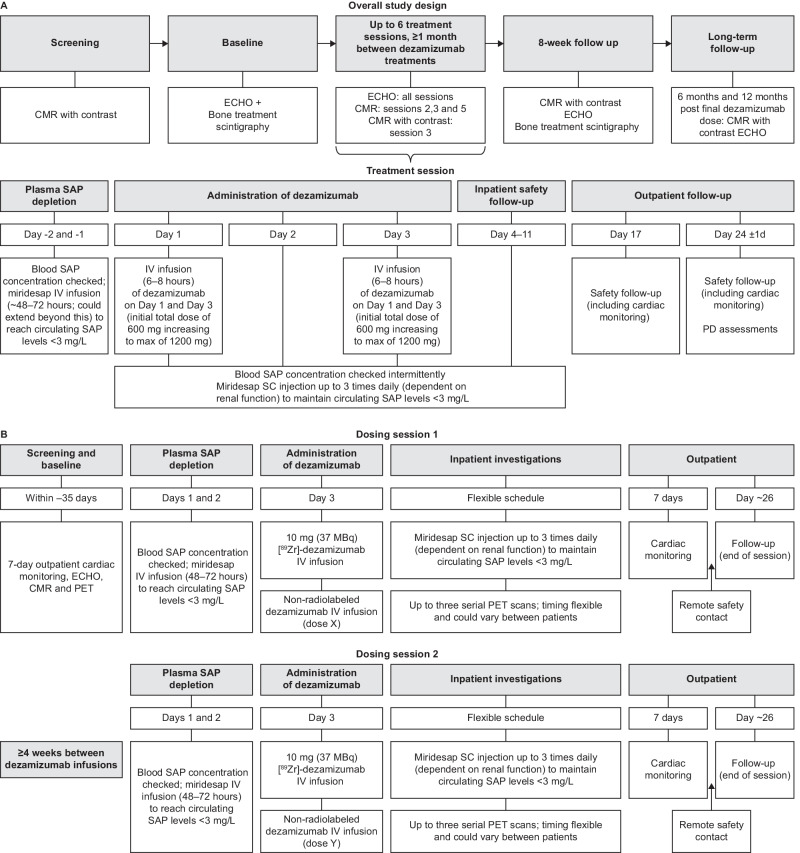
Study design for the Phase 2 study (**A**) and immuno-PET study (**B**). *Blood SAP depletion could extend beyond Days –2 and –1. *Note*: for the Phase 2 study the start of anti-SAP monoclonal antibody treatment was denoted as Day 1, whereas for the immuno-PET study the start of miridesap treatment was denoted as Day 1. *CMR* cardiac magnetic resonance, *ECHO* echocardiogram, *IV* intravenous, *MRI* magnetic resonance imaging, *PET* positron emission tomography, *SAP* serum amyloid P component, *SC* subcutaneous

The study consisted of screening and baseline sessions (conducted in a 6-week period prior to the first anti-SAP treatment), up to six anti-SAP treatment sessions with patients admitted as inpatients for the first 2 weeks of each cycle (minimum of 1 month between the start of each dezamizumab treatment), and 8-week, 6-month and 12-month follow-up sessions (Fig. 1a).

Each dosing session consisted of an initial depletion of circulating SAP to the target level by miridesap intravenous (IV) infusion (48 h over Days –2 and –1 but could have been administered up to 72 h; dose dependent on renal function) with maintenance of circulating SAP depletion on Day 1–11 by miridesap subcutaneous injection up to 3 times daily dependent on renal function. Dezamizumab 600 mg was selected as the initial dose to reduce the risk of haemodynamic complications from dezamizumab-associated infusion reactions in this cardiac patient population with left ventricular (LV) dysfunction. Dezamizumab was delivered in two split doses based on findings from the first-in-human study that indicated dezamizumab-associated skin rashes may be related to the maximum plasma concentration (C_max_) of the antibody [13]. Dose escalation on subsequent sessions was considered on a case-by-case basis up to a maximum of 1200 mg delivered as two infusions over 6–8 h each, on Days 1 and 3.

The first interim analysis was performed after five patients in Group 1 had completed at least three courses of anti-SAP treatment. Due to study termination, further planned interim analyses were not conducted.

#### Patient population

Patients were recruited from the UK and US (Additional file 1). Males and females aged 18–80 years with late-gadolinium enhancement on cardiac magnetic resonance (CMR) indicative of cardiac amyloidosis were eligible.

For Group 1, wild-type TTR patients were defined as having a cardiac or non-cardiac biopsy showing amyloid deposits that were confirmed as TTR-related by mass spectrometry or a positive technetium-dicarboxypropane diphosphonate or technetium-pyrophosphate scan in the absence of evidence of a plasma cell dyscrasia, and negative genetic screening for mutant TTR. Similar diagnostic criteria applied to those with hereditary ATTR-CM, except that they were required to have a known amyloidogenic TTR mutation recognised as primarily associated with CM demonstrated by genotyping. Patients were also required to be clinically stable (New York Heart Association [NYHA] class II or III) for 3 months pre-screening, with left ventricular mass (LVM) on CMR > 200 g.

For Group 2, patients with post-chemotherapy AL amyloidosis were required to have completed any line of chemotherapy or autologous stem cell transplantation at least 6 months previously and were required to have attained a very good partial response (reduction to < 40 mg/L in the difference in involved and uninvolved free light chain) or a complete response (normalisation of the free light chain levels and ratio, negative serum and urine immunofixation) [15]. AL amyloidosis was previously diagnosed by tissue biopsy in the setting of a plasma cell dyscrasia and appropriate amyloid typing. Patients were also required to be clinically stable (NYHA class II or III) for 3 months pre-screening with LVM on CMR > 150 g.

For Group 3, patients would have been required to have newly diagnosed AL amyloidosis and be Mayo stage II or IIIa with LVM on CMR > 150 g.

Patients were excluded from the study if any of the following criteria were met: corrected QT interval > 500 ms; history of sustained/symptomatic monomorphic or rapid polymorphic, ventricular tachycardia; unstable heart failure (emergency hospitalisation for worsening, or decompensated heart failure or syncopal episode) ≤ 1 month prior to screening.

Full inclusion and exclusion criteria are provided in the Additional file 2.

#### Endpoints

The primary endpoints of this study were a change in LVM over time from baseline to 8-week follow-up, and clinical safety data from AEs, clinical laboratory tests, vital signs, 12-lead electrocardiogram (ECG), cardiac monitoring and echocardiogram (ECHO) to 8-week follow-up, and incidence and grading of skin rashes classified using the Common Terminology Criteria for Adverse Events.

Secondary endpoints included investigation of the rash associated with anti-SAP treatment by histopathological and immunohistochemical examination of skin biopsies and blood biomarkers, characterisation of the pharmacokinetics (PK) of dezamizumab, evaluation of the changes to circulating biomarkers associated with pharmacodynamic (PD) effect, and evaluation of imaging markers associated with cardiac dysfunction by serial CMR and/or ECHO. Exploratory endpoints included change in cardiac extracellular volume (ECV) from baseline to 8-week follow-up. Further exploratory endpoints are listed in the Additional file 3.

#### Assessments

Multimodality cardiac imaging was carried out at pre-determined time points throughout the study, as shown in Fig. 1. All CMR and ECHO parameters were acquired and analysed by central imaging core laboratories [16, 17]. Parameters included, but were not limited to, strain, LV twist, stroke volume, ejection fraction, end diastolic volume, and the ratio between early mitral inflow velocity and mitral annular early diastolic velocity. All investigators and sponsor study team members, except those involved in central imaging core laboratory review, had access to the patient’s individual study treatment and dosing schedules for each constituent drug. Further ECHO imaging could be requested by the investigator at any time to monitor safety. CMR methodology is described in Additional file 4.

Safety assessments conducted throughout the Phase 2 study included 12-lead ECG, lead II telemetry and monitoring of vital signs. AEs were collected from the start of treatment until the 8-week follow-up. Blood samples for the analysis of drug concentration, biomarkers and PD markers were collected at various time points throughout the study*.* Markers analysed include complement markers and inflammatory markers (C-reactive protein [CRP]).

### Phase 1 immuno-PET study

#### Study design and treatment

This open-label, non-randomised, single-centre, two-part [^89^Zr]Zr-dezamizumab PET imaging study (NCT03417830) included clinically stable patients with cardiac dysfunction caused by ATTR-CM (Fig. 1b). It was conducted concurrently with the Phase 2 study described above. A detailed description of the methods can be found in Additional file 5. In short, following SAP depletion, patients received an IV infusion of non-radiolabelled dezamizumab plus a separate, concurrent IV infusion of [^89^Zr]Zr-dezamizumab. This was followed by up to three serial PET scans. The protocol was originally planned to have two parts: Part A, in which three patients were to receive up to two treatment sessions, and Part B, in which up to three patients were to receive one treatment session of ~ 26 days in duration. A formal interim analysis was planned to be conducted before progression to Part B, but the study was terminated prior to completion of Part A, and as a result, neither the formal interim analysis nor Part B were conducted.

#### Endpoints

The primary endpoint of the PET study was standardised uptake values (SUV; measured radioactivity concentration corrected for radioactive decay and normalised for administered amount of radioactivity per body weight) in focal anatomical locations within the heart and SUV of the whole heart at different time points after [^89^Zr]Zr-dezamizumab administration and at different dezamizumab total mass doses.

Secondary endpoints included focal and total radioactivity uptake in different tissues at different time points and after different total mass doses; descriptive PK parameters of total and radiolabelled dezamizumab; all AEs, including the incidence and grading of skin rashes, cardiac AEs, and infusion-related reactions; and other safety information, including ECG and vital signs. For exploratory objectives of this study, see the Additional file 3.

## Results

### Patient disposition

Due to early study termination, recruitment was lower than planned for both studies. The Phase 2 study enrolled seven patients (six patients in Group 1 and one patient in Group 2). A further five patients were screened but not randomised. In Group 1, six patients with ATTR-CM received 3–6 dosing sessions with total dezamizumab doses of 3000–6600 mg; all patients completed the study. In Group 2, one patient with AL received a partial dose of dezamizumab 300 mg on Day 1; as described in the safety section, this patient was withdrawn due to a serious AE (SAE).

The immuno-PET study recruited two patients with ATTR-CM. Patient A received two dosing sessions (9.94 mg radiolabelled and 70 mg of non-radiolabelled dezamizumab in session 1 and 9.83 mg labelled and 490 mg non-radiolabelled dezamizumab in session 2) and completed the study; Patient B received one dosing session (10.38 mg radiolabelled and 70 mg non-radiolabelled dezamizumab) and completed dosing session 1 but was withdrawn prior to starting session 2 due to the early study termination.

### Patient demographics and baseline characteristics

Baseline demographics and characteristics are presented in Table 1 for both studies. All patients were ≥ 65 years old. There was a noteworthy difference in the baseline LVM for the two patients enrolled in the immuno-PET study: 72 g for the first patient (Patient A) versus 298 g for the second patient (Patient B). Patient A’s diagnosis of ATTR-CM was based on a positive fat pad biopsy with both positive Congo Red and TTR staining, and typical changes of diastolic LV dysfunction on 2D-ECHO and a history of acute diastolic heart failure with NYHA class III clinical presentation that had improved to NYHA class II at study screening with the administration of diuretic therapy.

**Table 1:** Patient demographics and baseline characteristics

	Phase 2 study (Group 1 and 2) (n = 7)		Immuno-PET study (n = 2)
Sex, n (%)			
Male	7 (100)		1 (50)
Female	0 (0)		1 (50)
Mean (SD) age, years	73.3 (4.07)		77.5 (3.54)
Ethnicity			
Not Hispanic or Latino, n (%)	7 (100)		2 (100)
Race, n (%)			
White	7 (100)		2 (100)
Cardiac amyloidosis type, n (%)			
ATTR-CM	6 (85.7)		2 (100)^a^
AL	1 (14.3)		0 (0)
Mean (SD) cumulative miridesap dose, mg	Group 1 (n = 6)	12,519.3 (3245.35)	3394.0 (1406.61)
	Group 2 (n = 1)	1291.0	
Mean (SD) cumulative dezamizumab dose, mg	Group 1 (n = 6)	5379.5 (1498.67)	330.1 (353.1)
	Group 2 (n = 1)	300.0	

*ATTR-CM* transthyretin amyloidosis cardiomyopathy, *AL* immunoglobulin light chain amyloidosis, *PET* positron emission tomography, *SD* standard deviation

^a^ATTR-CM diagnosis not databased

### SAP depletion

In the Phase 2 study, depletion of circulating plasma SAP to target (< 3 mg/L) was always achieved prior to dezamizumab administration and was maintained throughout the testing period following dezamizumab administration. The same was true for the pre-dezamizumab levels in the PET study (circulating plasma SAP was not measured post-dezamizumab).

### Pharmacokinetics

In the Phase 2 study, exposure to dezamizumab was similar across patients and consistent across dosing sessions at the same dose level. Derived anti-SAP mAb PK parameters including C_max_ and area under the concentration–time curve until the last measurable concentration (AUC[0–t]) were stable from treatment session 2 in the Phase 2 study, and time to C_max_ (T_max_) was obtained within 57 h of first dezamizumab dose in all treatment sessions (Table 2). A double ‘spike’ profile was observed, consistent with the split dosing regimen, followed by a tail-off to low levels of detection at Day 11 and beyond in each treatment (Additional file 6). Characterisation of plasma PK of radiolabelled and non-radiolabelled dezamizumab in the immuno-PET study is presented in Additional file 7.

**Table 2:** Geometric mean (95% CI) plasma PK of dezamizumab in Group 1^a^ of the Phase 2 study (safety population)

PK parameter^b^	Session 1 (n = 6)	Session 2 (n = 6)	Session 3 (n = 6)	Session 4 (n = 5)	Session 5 (n = 4)	Session 6 (n = 4)
AUC_(0–t)_, h*μg/mL	5130 (3218, 8178)	14,610 (10,253, 20,820)	13,805 (8383, 22,732)	12,971 (7101, 23,692)	15,595 (7244, 33,574)	17,127 (8772, 33,440)
C_max_, µg/mL	86.94 (62.90, 120.16)	235.48 (181.46, 305.57)	222.23 (142.39, 346.83)	179.82 (119.43, 270.73)	185.52 (109.51, 314.31)	228.51 (108.15, 482.82)
T_max_, h	54.23 (53.88, 54.58)	53.95 (53.40, 54.50)	42.43 (22.27, 80.82)	54.74 (53.64, 55.87)	31.62 (5.47, 182.82)	54.56 (53.06, 56.11)

^a^Data from the one session in Group 2 were: AUC_(0–t)_ 2148 h*µg/mL; C_max_ 88.64 µg/mL; T_max_ 6.07 h

^b^Anti-SAP mAb was administered on Day 1 and Day 3 of the treatment session as a 300 mg dose on each day in session 1 and as a 600 mg dose on each day in subsequent sessions, with the exception of one patient from session 3 onwards where dose was split 300 mg:600 mg. The Group 2 patient received only one dose of 300 mg in the first session prior to treatment termination

AUC_(0–t)_, area under the concentration–time curve until the last measurable concentration; *CI* confidence interval, *C*_*max*_ maximum concentration, *PK* pharmacokinetics, *T*_*max*_ time to reach C_max_

### Pharmacodynamic markers and immunogenicity (Phase 2 study)

A mean increase from pre-treatment baseline in high-sensitivity CRP and serum amyloid A protein (SAA) was evident from Day 2 (Group 2) and Day 3 (Group 1) of treatment session 1 and, for Group 1, in subsequent sessions, indicating an inflammatory response following anti-SAP mAb treatment. High-sensitivity CRP increased in the range 0.3–64.3 mg/L from Day 3 for Group 1 (e.g. Session 2, Day 3, 8H time point: Mean (SD) 30.75 (24.329) mg/L), and to 41.4 mg/L from Day 2 for the patient in Group 2. SAA values for Group 1 from Day 3 increased in the range 0.2314–430.4676 mg/L; for the patient in Group 2, SAA increased to 322.2895 mg/L from Day 2.

**Fig. 2:**
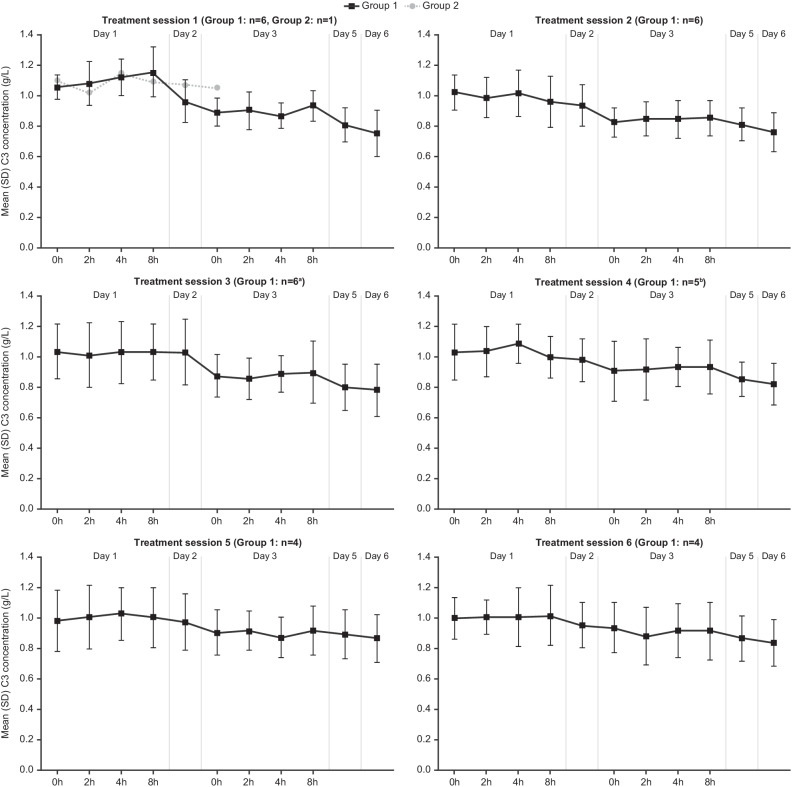
Mean (SD) complement C3 concentration over time (Phase 2 study; safety population). **A** n = 5 on Day 2; **B** n = 4 at the 4-h time point on Day 1. *SD* standard deviation

In addition, an observed reduction in plasma complement markers (notably C3 and CH50), with a return to near pre-baseline ranges before initiation of the next treatment session. In the Phase 2 study, mean reductions from pre-treatment baseline were observed for complement C3 values at most time points during each of the six treatment sessions for patients from Group 1. The largest mean reductions were observed by Day 5 or 6 within each treatment session (which were the last days of C3 measurement). Mean pre-dezamizumab values of C3 at the start of each treatment session had returned to a similar range to that observed at pre-treatment baseline (Fig. 2).

This is potentially indicative of target (SAP) engagement but it was not possible to discern the localisation of anti-SAP mAb binding in this study, and whether this was successfully achieved for target organ amyloid deposits.

An immunological response to anti-SAP mAb was evident by the presence of anti-drug antibodies at the 8-week follow-up (designated as positive or negative), with continued long-term positive assessments at 6-month follow-up visits for all patients who had received a minimum of four treatment sessions (data not shown). The only patient who did not display a positive anti-drug antibody result at these assessment times received only three treatment sessions.

### LVM, LV thickness, and ECV (Phase 2 study)

**Fig. 3:**
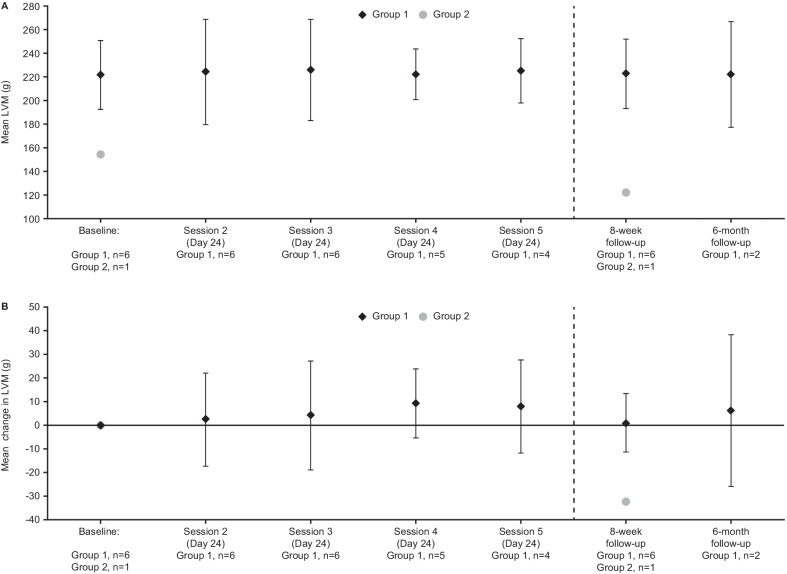
**a** Mean (SD) LVM over time; **b** mean (SD) change from baseline in LVM over time (Phase 2 study; safety population). Vertical bars show SD. *LVM* left ventricular mass, *SD* standard deviation

In Group 1 of the Phase 2 study, there was no evidence of cardiac amyloid removal over time as measured by the primary endpoint of reduction in LVM by CMR (Fig. 3; patient-level data are shown in Additional file 8). Small mean increases in LVM were observed at all post-baseline time points for patients with ATTR-CM, although at the 8-week follow-up visit the patient from Group 2, who had a lower baseline LVM than all Group 1 patients, had a small reduction from pre-treatment baseline in LVM (change from baseline: − 32.42 g). There was no consistent mean change from baseline in LV wall thickness at any time point as monitored by ECHO. The exploratory endpoint of ECV measured on CMR showed no consistent change from baseline at the 8-week follow-up (ranging from a 7.27% decrease to a 6.61% increase).

### Cardiac structural and functional endpoints (Phase 2 study)

At the 8-week follow-up time point, individual changes from baseline in cardiac functional measures for patients from Group 1 were variable. In addition, results were not always directionally consistent across CMR and ECHO at this time point where both methods were utilised; for example, LV end diastolic volume and LV stroke volume parameters showed a mean increase from baseline for the primary CMR assessment and a mean decrease from baseline when using ECHO. A small decrease from baseline in LV ejection fraction (%) was observed at 8-week follow-up using CMR and ECHO (Additional file 9).

Absolute NT-proBNP values at baseline ranged from 1631 to 5020 ng/L across patients from Group 1 with a lower value of 444 ng/L for the patient from Group 2. The corresponding baseline values for Troponin T (high sensitivity) were 32–89 ng/L (Group 1) and 27 ng/mL (Group 2). There was no consistent pattern of change from baseline in NT-proBNP values across patients or treatment sessions. Change from baseline values at the 8-week follow-up ranged from a 1067 ng/L decrease to a 2442 ng/L increase. For patients from Group 1 (with high-sensitivity assays performed), Troponin T values showed increases from baseline for most patients from Day 5 in session 4 and beyond. Increases from baseline values at the 8-week follow-up ranged from 6 to 67 ng/L.

### Cardiac uptake and biodistribution

**Fig. 4:**
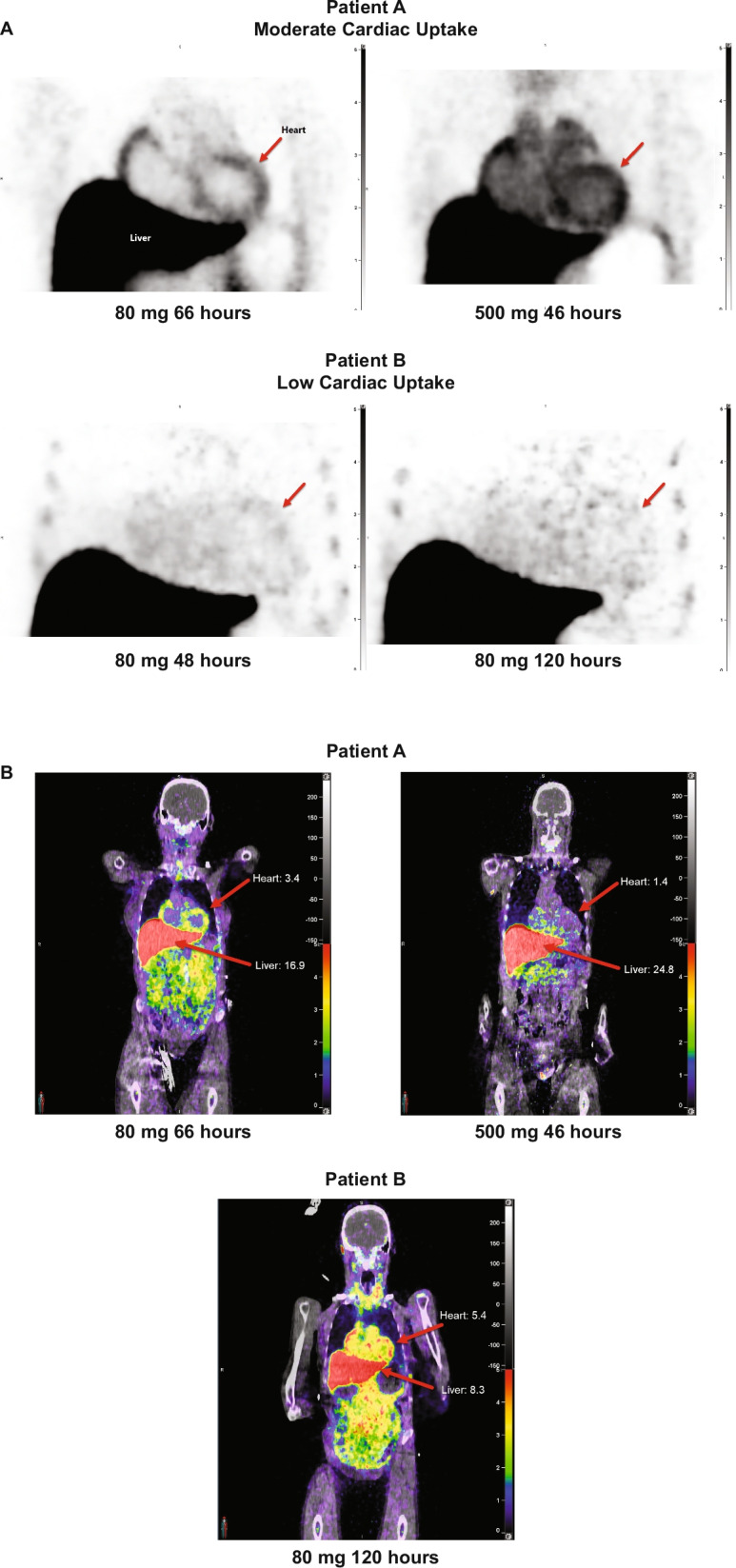
**A** Coronal view of cardiac uptake of [^89^Zr]Zr-dezamizumab and **B** whole-body standardised uptake values (immuno-PET study; all-treated population)

For the PET study, the study protocol specified that dezamizumab was considered to have reached the extracellular space of cardiac muscle if the cardiac-tissue-to-plasma ratio was ≥ 0.5 at any time point or any session for a given patient; this value was chosen as it is appreciably higher than the expected contribution of 0.1–0.2 from the interstitium. The uptake of [^89^Zr]Zr-dezamizumab in various anatomical locations within the cardiac muscle was moderately high in Patient A, who had the lower LVM, with SUV peak values mostly in the range of 2–5 (a value of 1 indicates even distribution in the body). Uptake in the cardiac muscle was also seen, but was generally lower in Patient B, who had the higher LVM (Table 3; Fig. 4). For Patient A, who participated in two dosing sessions, on the only comparable day between sessions (Day 4) a slight increase was seen in cardiac muscle SUV in the second session. The corresponding blood pool values were also slightly higher (Table 3).

**Table 3:** [^89^Zr]Zr-dezamizumab biodistribution in cardiac and non-cardiac tissues/organs expressed as SUV over time (immuno-PET study; all-treated population)

	Patient A					Patient B		
	Session 1		Session 2			Session 1		
	Day 4	Day 6	Day 3	Day 4	Day 5	Day 4	Day 5	Day 8
Organ/tissue	SUV mean							
Liver	12.96	16.94	4.77	7.56	8.34	22.65	25.56	24.80
Adrenal	13.03	12.02	4.80	8.38	6.36	10.30	10.77	10.90
Spleen	9.80	10.06	4.44	5.14	5.44	13.05	11.05	11.34
Thyroid—goiter hotspot	4.67	4.24	1.89	3.69	3.19	–	–	–
Kidney	4.08	3.84	2.70	3.26	2.89	5.74	5.13	4.82
Testes	–	–	–	–	–	5.40	5.52	5.67
Aorta	3.56	0.50	10.00	5.89	3.31	1.60	0.36	0.65
Heart	3.30	1.32	7.09	4.44	3.39	1.64	1.13	0.96
Bone marrow	2.45	2.21	1.86	1.91	1.41	3.58	3.17	3.88
Cardiac focal anatomical locations	SUV peak							
Blood pool left atrium	4.03	0.64	10.29	5.33	3.55	1.96	0.55	0.40
Blood pool left ventricle	3.88	0.85	10.12	4.91	3.11	1.72	0.51	0.43
Blood pool right ventricle	3.98	0.95	10.39	5.16	3.50	2.35	1.05	1.24
Left ventricle wall—high uptake	3.40	3.40	1.15	4.54	5.36	1.36	1.29	1.44
Left ventricle wall—low uptake	2.76	1.84	3.11	3.65	3.98	0.72	0.70	0.76
Mid septum—high uptake	3.22	2.88	4.68	4.20	4.78	1.45	1.42	1.52
Mid septum—low uptake	3.09	2.23	3.58	3.57	3.80	0.82	0.78	0.61

[^89^Zr]Zr-]-dezamizumab administered on Day 3

*PET* positron emission tomography, *SUV* standardised uptake values

Regarding overall biodistribution in the PET study, dezamizumab was principally distributed to multiple abdominal organs (Table 3; Fig. 4). At session 1, the biodistribution of [^89^Zr]Zr-dezamizumab was highest in the liver, adrenal gland and spleen for both patients, followed by the kidney and testes for Patient B and thyroid goiter hotspot for Patient A (Table 3). For Patient A, who participated in two dosing sessions, the uptake of [^89^Zr]Zr-dezamizumab in the high-uptake organs (liver, spleen and adrenals) was in general lower in session 2, and specifically on the only comparable day between sessions (Day 4) (Table 3).

### Safety

No deaths were reported in either study. In the Phase 2 study, all patients reported at least one AE during the treatment phase, and all but one patient had a treatment-related AE (Table 4). One treatment-related SAE was reported and is described further below. There were no clinically significant findings from laboratory tests, vital signs, cardiac monitoring, ECHO and ECG assessments. In two patients from Group 1, ventricular tachycardia and extrasystoles in a frequency higher than seen at baseline were reported on Session 1 Day 5 and Session 3 Day 4 for one patient and on Session 1 Day 3, Session 2 Day 10 for the other. This latter patient also had increased frequency of ventricular extrasystoles on Session 2 Day 9.

**Table 4:** Summary of AEs in the Phase 2 study (safety population)

AE, number of participants (%)^b^	Anti-SAP treatment^a^	
	Group 1 (n = 6)	Group 2 (n = 1)
At least one AE	6 (100)	1 (100)
Related AE^c^	5 (83)	1 (100)
Leading to withdrawal from study/permanent discontinuation of anti-SAP treatment	0	1 (100)
SAEs	1 (17)	1 (100)
Fatal	0	0
Related to anti-SAP treatment	0	1 (100)^d^
Rash category events (maximum CTCAE grade)^e^		
Any	4 (67)	0
Grade 1	3 (50)	0
Grade 2	1 (17)	0
Temporally associated, potential mAb-related infusion events^f^	4 (67)	1 (100)
Medically confirmed infusion-related reactions	1 (17)^d^	0

^a^Anti-SAP treatment could be dezamizumab and/or miridesap

^b^Only includes AEs assigned to the on-treatment phase, i.e. with AE start date/time between first dose date/time of study drug and last dose date/time of study drug + 56 days, inclusive

^c^Related to dezamizumab or miridesap

^d^Related to dezamizumab

^e^AEs categorised as RASH included “rash”, ‘maculopapular rash’, ‘pruritus’, and ‘rash erythematous’. Only those regarded as related to dezamizumab treatment in the opinion of the investigator were included and categorised using the CTCAE grading system

^f^Temporally associated potential mAb-related infusion reactions (TPMI) were determined programmatically using the following preferred terms: ‘headache’, ‘flushing’, ‘feeling hot’, ‘feeling cold’, ‘chest discomfort’, ‘chills’, ‘face oedema’, ‘oedema peripheral’, ‘orbital oedema’, ‘nausea’, ‘vomiting’, ‘diarrhoea’, ‘fatigue’, ‘tachycardia’, ‘presyncope’ and ‘infusion-related reaction’. Events with these terms that occurred during the mAb infusion or within 24 h of the end of the mAb infusion were regarded as TPMI events. All TPMI events were medically reviewed to determine infusion-related reactions

*AE* adverse event, *CTCAE* Common Terminology Criteria for Adverse Events, *mAb* monoclonal antibody, *SAE* serious adverse event, *SAP* serum amyloid P component

In the immuno-PET study, both patients experienced AEs. There were a total of eight AEs, four of which (reported in two patients) were considered treatment-related: headache, dactylitis, injection-site bruising and urticarial vasculitis (moderate grade 2 skin rash) (Table 5). Each patient experienced at least one treatment-related AE (i.e. related to dezamizumab and/or miridesap, not related to [^89^Zr]Zr-dezamizumab). There were no cardiac AEs, cardiac SAEs or AEs leading to treatment discontinuation. There were no laboratory values or vital signs of potential clinical importance. Both patients had ECGs and telemetry assessments that were classed as ‘abnormal – not clinically significant’ before and after treatment.

**Table 5:** Summary of on-treatment AEs in the immuno-PET study (safety population)

Preferred term	Patients, n (%) N = 2	AEs, n N = 2	Drug-related AEs, n (%) N = 2
Any AE	2 (100)	8	2 (100)
Headache	2 (100)	2	1 (50)
Dactylitis	1 (50)	1	1 (50)
Diarrhoea	1 (50)	1	0
Dry eye	1 (50)	1	0
Injection-site bruising	1 (50)	1	1 (50)
Restless legs syndrome	1 (50)	1	0
Urticarial vasculitis	1 (50)	1	1 (50)

*AE* adverse event, *PET* positron emission tomography

In both studies dezamizumab was associated with development of rash, which in the Phase 2 study was the most frequently reported AE during the treatment phase. Rash events deemed related to treatment were reported by four patients in the Phase 2 study and one patient in the immuno-PET study. The incident rashes were characterised as urticarial and/or maculopapular in nature. The rash AEs were grade 1 or 2. Grade 1 rashes were treated with topical steroids, antihistamine and/or analgesic as required. One patient in each trial developed a grade 2 rash; the patient in the Phase 2 trial received a reduced dose of dezamizumab on subsequent treatment sessions. Histopathological review of rash-associated skin biopsies obtained in two patients in the Phase 2 study showed leukocytoclastic vasculitis (LCV) involving small dermal vessels in the superficial dermis. The neutrophil-dependent mechanism of LCV was confirmed by strong positive neutrophil-specific elastase staining in skin rash biopsies versus negative staining in baseline biopsies (data not shown). A biopsy performed in the one patient with urticarial vasculitis in the PET study also was reported to show LCV.

In the Phase 2 study, on Day 1 of the first session in Group 2 (post-chemotherapy AL amyloidosis), an unexpected serious adverse reaction of presumptive abdominal large-vessel vasculitis that was considered related to treatment was reported following administration of dezamizumab 300 mg. The patient experienced acute abdominal pain and bloating within 24 h of drug administration. Systemic inflammatory markers were elevated, including CRP and white blood cell count with neutrophilia but no eosinophilia. There was new onset ﻿﻿haematuria which resolved by Day 24, though there was no skin rash, and antinuclear bodies/antineutrophil cytoplasmic antibodies were negative. An abdominal CT scan revealed thickening of abdominal large arterial vessels (aorta, both iliac and renal arteries) and fat stranding in the mesentery. Symptoms were mostly resolved within 24 h of a single IV dose of methylprednisolone 1 g. An [^18^F] fluorodeoxyglucose (FDG)-PET scan performed 72 h after administration of methylprednisolone revealed no discernible increased [^18^F]FDG uptake in any large arterial vessel, which was consistent with the intended anti-inflammatory effect of high-dose steroid rescue, and prompt resolution of the patient’s abdominal symptoms. The event was reported as resolved within 16 days following methylprednisolone treatment. Ongoing clinical surveillance demonstrated no acute or residual effects on biochemical renal function or urine output; a follow-up CT scan 8 weeks later demonstrated near complete anatomical resolution of abdominal aortic wall thickening. Based on the totality of the information, the investigator and vascular radiologist concluded that the patient experienced extensive large- and small-vessel vasculitis related to the study drug, which was substantially resolved.

## Discussion

In this manuscript we present data from two concurrent studies in patients with cardiac amyloid treated with dezamizumab, a mAb to SAP, following SAP depletion with miridesap. The Phase 2 study assessed the magnitude and time course of amyloid removal in the heart in patients with either ATTR or AL amyloidosis, as measured by CMR. The PET imaging study provided direct assessment of the exposure of the cardiac tissue to dezamizumab as well as its wider biodistribution, strengthening the understanding of exposure versus therapeutic evidence that was assessed in the Phase 2 trial.

The principal finding of the Phase 2 study was a lack of evidence of cardiac amyloid removal over time with anti-SAP treatment. Anti-SAP treatment was well tolerated by the six subjects with ATTR-CM, although a rash was identified as the most commonly reported AE. The one AL subject in the trial reported a SAE of large-vessel vasculitis, unexpected from the previous known profile. The occurrence of this event in conjunction with lack of cardiac signal of efficacy led to reconsideration of the benefit-risk assessment for the treatment. The principal finding of the concurrent PET imaging study was the demonstration of only low to moderate uptake of radioactive-labelled anti-SAP mAb in the cardiac tissue at the doses studied, provided supportive evidence of the basis for the Phase 2 findings.

Prior evaluation of this anti-SAP treatment demonstrated amyloid removal in patients with liver, spleen and kidney involvement [13, 14], and a link was seen between reduction in hepatic amyloid load and improvement in liver function [14]. However, our assessment of treatment impact on cardiac involvement failed to provide consistent evidence of cardiac amyloid removal or functional benefit. In particular, the exploratory endpoints of ECV, native T1 mapping, late gadolinium enhancement, and global longitudinal strain, all considered to be of prognostic utility in this disease, showed no evidence of favourable changes with treatment. If miridesap/dezamizumab, with the dosing regimens used, was effective in removing cardiac amyloid, we would expect to see a reduction in LVM within the study period based on the time frame for reductions in amyloid load that were observed in Phase 1; this reduction would be in contrast to amyloid regression with treatments that reduce the precursor source and that take several years to show an effect [18].

The decision for an early termination of the study was based on the lack of evidence of cardiac amyloid removal or functional benefit, and the reporting of a SAE of vasculitis in a subject in the Phase 2 study. The conclusion of the safety evaluation was that the risk of exposing further subjects to anti-SAP treatment outweighed the potential for benefit observed in the study.

The immuno-PET study data are severely limited given enrolment of just two patients. In the study protocol, cardiac uptake was defined as a cardiac-tissue-to-plasma ratio > 0.5 at any time point or any session, which for a haematocrit of approximately 0.5, equates to a tissue-to-blood ratio > 1.0. If there was only a vascular contribution to the signal in the heart, the tissue-to-blood ratio of the myocardium would be expected to be < 0.1 [19]. By this definition, dezamizumab was shown to be able to access the interstitial space, although at best the uptake was moderate to low. A high uptake could conceivably be possible if there was good tissue access and a significant binding to SAP–amyloid complexes. Furthermore, such high uptake could diminish in a second study due to competition by a high amount of non-radiolabelled dezamizumab. Cardiac uptake was seen in both dosing sessions in Patient A but uptake in various anatomical locations within the myocardium was limited in comparison to liver and spleen, with most SUV peak values in the range of 2–5. In Patient B, who was the more typical ATTR-CM patient, only one dezamizumab dose was received and only minor uptake was seen in the LV septum. These limited data suggest that, although dezamizumab does have the ability to be taken up into the myocardial interstitium, it is at a low level with the dosing regimen tested and with a significant ‘sink’ effect, i.e. preferential antibody binding in non-cardiac organs (primarily, but not limited to, liver and spleen). The mechanisms behind the high accumulation of radiolabelled antibody in these organs is not clear, but a noteworthy aspect is that the uptake in these organs is proportionally decreased at the higher mass dose in Patient A. The effect likely had a significant impact on the degree of cardiac penetration, and it is unknown whether longer administration would lead to increased cardiac penetration, once the peripheral sink is saturated. Of note, the different myocardial uptake profiles in the two wild-type ATTR-CM patients enrolled may be a result of the different features of their disease as evidenced by their LVM values: 72 g for Patient A, and 298 g for Patient B. It is possible that Patient A was in earlier stages of cardiac amyloidosis, or that the CM may be (at least partially) attributed to other causes, such as pre-existing diabetes, whereas Patient B had more pronounced cardiac amyloid deposition.

Coronary microvascular perfusion is known to be impaired in TTR amyloidosis. Thus, one additional reason for the discriminatory imaging findings between the two patients in the immuno-PET study is that since LVM is indicative of the extent of amyloid in the interstitial space of the myocardium, extensive amyloid infiltration in Patient B could have led to severe impairment in microvascular perfusion of the myocardium, leading to limited dezamizumab distribution to the heart. This is further supported by the finding in the Phase 2 study that all the ATTR-CM patients who had a similar cardiac amyloid mass to Patient B in the PET study had low coronary perfusion parameters at rest on baseline magnetic resonance imaging (data not shown). However, further correlative pathological imaging research would be needed to confirm the pathophysiological basis for the poor cardiac distribution of dezamizumab and the difference in distribution between the two patients.

In the previous first-in-human study, evidence of amyloid clearance was generally preceded by a fall in C3 (and to a lesser extent C4) [13, 14]. This is consistent with the mechanism of action of dezamizumab [12]. In the Phase 2 study presented here, there was an observed reduction in plasma total C3 levels with a return to the near pre-baseline range before initiation of the next treatment session. Although these findings are indicative and potentially sentinel for dezamizumab–SAP engagement, we are unable to comment on the quality of immunological engagement since the depth of circulating C3 activation was not evaluated by measurement for plasma C3 activation products. As such, since we could not evaluate the precise tissue localisation of dezamizumab–SAP binding (I.e. amyloid deposits vs normal tissue), we cannot provide a complete PD characterisation of dezamizumab in patients with cardiac amyloidosis.

It is possible that the observed reduction in plasma total C3 levels represents PD activity in extra-cardiac organs. The preliminary results of the PET study suggest that while dezamizumab can enter cardiac tissue the amounts are small, which provides a rationale for the lack of impact of dezamizumab on cardiac amyloid load (LVM) or the functional parameters explored in the Phase 2 study. Although amyloid reduction was demonstrated in the first-in-human study in other visceral organs, the necessary triggering of the PD response for removal of amyloid fibrils in the heart is not possible without adequate delivery of the mAb to cardiac tissue to achieve a sufficient density of binding of mAb to cardiac amyloid deposits. In addition to impaired cardiac microvascular perfusion, this may also be a result of the continuous structure of the endothelium in cardiac tissue in contrast to the fenestrated endothelium found in the liver and spleen, the organs in which the most striking treatment effects had previously been seen triggering of a complement-dependent macrophage giant cell response by dezamizumab is dependent on achieving a sufficient density of binding of mAb to target, and the amount of mAb binding to SAP in cardiac tissue may have been insufficient to trigger the response. Alternative explanations could be inadequate entry of complement or macrophage/monocytes for similar reasons. Finally, the depletion of circulating SAP by miridesap necessary for the safe administration of dezamizumab also has been shown to remove SAP from amyloid deposits. It is therefore possible that the extent to which SAP was lowered in these studies affected the extent to which cardiac entry of dezamizumab could achieve target engagement and initiate amyloid removal.

SAP is also present in some vascular basement membranes and is part of the microfibrillar mantle of elastic fibres in normal human tissue [20–22]. The potential binding of the mAb to this SAP is the basis of dermal LCV manifesting as rash. Rash was common across these studies and phenotypically similar to that observed in the Phase 1 study. Crucially, this was mainly grade 1 or 2. The single patient with AL enrolled in the Phase 2 study (who had almost exclusively clinically-determined cardiac amyloid), reported an index event of suspected large abdominal vessel vasculitis. In considering the event to be large-vessel vasculitis/aortitis, we acknowledge that CT angiographic imaging has not previously been reported in patients with systemic amyloidosis in patients treated with miridesap and dezamizumab in the current studies or the Phase 1 study. We therefore cannot be sure whether the abnormalities, which were consistent with vasculitis, actually reflect that pathology or indeed whether vasculitis is a more frequent, if asymptomatic, occurrence. There is the potential for overlap between dermatological manifestations of LCV and an event of large-vessel vasculitis, as it is possible for vasculitis to affect small arteries [23]. However, the risk of large-vessel vasculitis is a significant concern due to potential catastrophic clinical implications (such as cerebrovascular, myocardial, or mesenteric ischaemia), and the potential for varied and sometimes vague clinical presentation (as in the index case). These implications (e.g. severity of sequelae, difficulty in monitoring, and lack of clear markers predicting its occurrence) led to the conclusion that the risk of exposing further patients to anti-SAP treatment was not justifiable given the lack of evidence for potential benefit from the Phase 2 interim data (i.e. no reductions in amyloid in the heart nor improvement in cardiac function) and the low cardiac update demonstrated in the PET study.

While the Phase I study showed finding suggesting that anti-SAP therapy can remove amyloid for other organs [14], the current data show that the dosing used in these studies we report is inadequate to target the myocardium. The PET study did not identify localisation of anti-SAP mAb binding in target organ and therefore localisation to other organs cannot be ruled out as an explanation for the lack of change in cardiac measures. Despite these disappointing results in terms of cardiac therapy, we believe that this experience has furthered insight into the potential pathophysiological challenges of delivering anti-amyloid therapy to the heart, an organ in which the effect of amyloid infiltration influences both myocardial structure and perfusion [24, 25].

The main limitation of these studies was the low sample number, with six patients completing the Phase 2 study, and one patient completing the immuno-PET study. Although the initial sample sizes were consistent with the objectives of the studies, sample sizes were further reduced by the early termination of the studies. Furthermore, there was no control data available from the Phase 2 study to quantify anticipated increases in cardiac amyloid attributable to the natural time course of the disease during the study period and hence interpretation of this finding is limited.

## Conclusions

In summary, the encouraging Phase 1 results with miridesap/dezamizumab showing removal of amyloid in several organs provided proof of mechanism to conduct the Phase 2 study in cardiac amyloidosis, the major determinant of prognosis in the two most common forms of systemic amyloidosis (ATTR and AL). Recognising the differences in antibody access to the myocardial interstitium compared with liver or spleen, we also conducted an exploratory PET study in parallel to confirm cardiac localisation. Data from the Phase 2 study from seven patients after up to six treatment sessions showed no convincing evidence of amyloid removal with the systemic doses of antibody used. The concurrent immuno-PET study showed only moderate to low uptake in the heart, while inflammatory responses observed in skin and vascular tissue, including the event of large abdominal vessel vasculitis in the Phase 2 study, suggested non-cardiac binding. With the unexpected observation of large-vessel vasculitis, it was considered unwise to test higher doses of dezamizumab in an attempt to deliver more antibody to the heart due to the potential increase in the risk of on-target off-amyloid binding. Given the findings of these studies, we considered the benefit-risk in cardiac amyloid was no longer favourable; therefore, the studies and the development of this treatment for cardiac amyloidosis were terminated.

## Supplementary Information

**Additional file 1**. Patient population details.

**Additional file 2.** Full inclusion and exclusion criteria.

**Additional file 3.** Study objectives.

**Additional file 4.** Phase 2 CMR methodology.

**Additional file 5.** Phase 1 immuno-PET study methods.

**Additional file 6.** Median plasma dezamizumab concentration–time plots by treatment cycle (Phase 2 study; safety population).

**Additional file 7.** Plasma PK of [^89^Zr]Zr-dezamizumab and total dezamizumab (non-radiolabelled dezamizumab and [^89^Zr]Zr-dezamizumab (immuno-PET study; PK population).

**Additional file 8**. Changes in LVM across treatment sessions for each patient (Phase 2 study).

**Additional file 9.** Summary of imaging markers of cardiac dysfunction as measured by CMR and/or ECHO imaging over time (safety population).Summary of imaging markers of cardiac dysfunction as measured by CMR and/or ECHO imaging over time (safety population).

## Data Availability

Anonymized individual participant data from this study plus the annotated case report form, protocol, reporting and analysis plan, data set specifications, raw dataset, analysis-ready dataset, and clinical study report are available for research proposals approved by an independent review committee. Proposals should be submitted to www.clinicalstudydatarequest.com. A data access agreement will be required.

## Abbreviations

AA: Amyloid A protein
AE: Adverse event
AL: Light chain
AUC: Area under the concentration–time curve
CI: Confidence interval
CM: Cardiomyopathy
C_max_: Maximum plasma concentration
CMR: Cardiac magnetic resonance
CRP: C-reactive protein
CT: Computed tomography
ECHO: Echocardiogram
ECG: Electrocardiogram
ECV: Extracellular volume
FDG: Fluorodeoxyglucose
IV: Intravenous
LCV: Leukocytoclastic vasculitis
LVM: Left ventricular mass
mAb: Monoclonal antibody
NT-proBNP: N-terminal pro-B-type natriuretic peptide
NYHA: New York Heart Association
PD: Pharmacodynamic
PET: Positron emission tomography
PK: Pharmacokinetic
SAA: Serum amyloid A protein
SAE: Serious AE
SAP: Serum amyloid P
SUV: Standardised uptake values
T_max_: Time to C_max_
TTR: Transthyretin
