# Supplementary material for: Pharmacodynamic evaluation and safety assessment of treatment with antibodies to serum amyloid P component in patients with cardiac amyloidosis: an open-label Phase 2 study and an adjunctive immuno-PET imaging study
Source: BMC Cardiovasc Disord. 2022 Feb 13;22:49. doi: 10.1186/s12872-021-02407-6 (PMC8843022; doi:10.1186/s12872-021-02407-6)
Supplement: Supplementary file 1 — Additional file 1. Patient population details. [file 12872_2021_2407_MOESM1_ESM.docx]

# additional file 1

# Patient population

Patients with cardiac amyloidosis were recruited from the UK and US (Phase 2 study), and Sweden (PET study). In the UK, patients were referred centrally from the National Amyloidosis Centre in London to two separate dosing sites at either the GSK Clinical Unit Cambridge at Addenbrooke’s Hospital, Cambridge, or Hammersmith Medical Research in London. In the US, patients were referred, recruited and treated at the Brigham and Women’s Hospital in Boston. In Sweden, patients were referred, recruited and treated at Clinical Trial Consultants AB in Uppsala.
