# Supplementary material for: Pharmacodynamic evaluation and safety assessment of treatment with antibodies to serum amyloid P component in patients with cardiac amyloidosis: an open-label Phase 2 study and an adjunctive immuno-PET imaging study
Source: BMC Cardiovasc Disord. 2022 Feb 13;22:49. doi: 10.1186/s12872-021-02407-6 (PMC8843022; doi:10.1186/s12872-021-02407-6)
Supplement: Supplementary file 2 — Additional file 2. Full inclusion and exclusion criteria. [file 12872_2021_2407_MOESM2_ESM.docx]

# Additional file 2

# Full inclusion and exclusion criteria

## Phase 2 study

### Inclusion criteria

Age

- Between 18 and 80 years of age inclusive, at the time of signing the informed consent.

Sex

- Both male and female subjects are eligible to participate.
- Males:
  - Male subjects with female partners of childbearing potential must comply with the following contraception requirements from the time of first dose of study. medication for a cycle of spermatogenesis following five terminal half-lives after the last dose of study medication.
    - a. Vasectomy with documentation of azoospermia.
    - b. Male condom plus partner use of one of the options:
      - Contraceptive subdermal implant
      - Intrauterine device or intrauterine system
      - Combined oral contraceptive or injectable progestogen
      - Contraceptive vaginal ring
      - Percutaneous contraceptive patches.
        - These allowed methods of contraception are only effective when used consistently, correctly and in accordance with the product label.
        - For non-product methods (eg, male sterility), the Investigator determines what is consistent and correct use.
        - The Investigator is responsible for ensuring that subjects understand how to properly use these methods of contraception.
- Females
  - A female subject is eligible to participate if she is not pregnant (as confirmed by a negative urine human chorionic gonadotrophin test), not lactating, and at least one of the following conditions applies:
    - Non-reproductive potential defined as:
      - Pre-menopausal females with one of the following:
        - Documented tubal ligation
        - Documented hysteroscopic tubal occlusion procedure with follow-up
        - Confirmation of bilateral tubal occlusion
        - Hysterectomy
        - Documented bilateral oophorectomy.
      - Postmenopausal defined as:
        - ≥60 years of age
        - Twelve (12) months of spontaneous amenorrhea with an appropriate clinical profile, eg, age appropriate, >45 years, in the absence of hormone replacement therapy (HRT) or medical suppression of the menstrual cycle (eg, leuprolide treatment) in questionable cases a blood sample with simultaneous follicle-stimulating hormone (FSH) and estradiol levels consistent with menopause (refer to laboratory reference ranges for confirmatory levels). Females on HRT and whose menopausal status is in doubt will be required to use one of the highly effective contraception methods if they wish to continue their HRT during the study. Otherwise, they must discontinue HRT to allow confirmation of postmenopausal status prior to study enrollment.
    - Reproductive potential and agrees to follow one of the following options:
      - Contraceptive subdermal implant
      - Intrauterine device or intrauterine system
      - Combined estrogen and progestogen oral contraceptive
      - Injectable progestogen
      - Contraceptive vaginal ring
      - Percutaneous contraceptive patches
      - Male partner sterilization with documentation of azoospermia prior to the female subject's entry into the study, and this male is the sole partner for that subject
      - The documentation on male sterility can come from the site personnel’s: review of subject’s medical records, medical examination and/or semen analysis, or medical history interview provided by her or her partner.
        - These allowed methods of contraception are only effective when used consistently, correctly and in accordance with the product label. The Investigator is responsible for ensuring that subjects understand how to properly use these methods of contraception.

Informed consent

- Capable of giving written informed consent, which includes compliance with the requirements and restrictions listed in the consent form.

Other

- Late-gadolinium enhancement on cardiac magnetic resonance (CMR) indicative of cardiac amyloidosis.

#### Inclusion criteria for Group 1

Type of subject and diagnosis including disease severity:

- Transthyretin (ATTR)-cardiomyopathy (ATTR-CM).
  - Subjects with a diagnosis of hereditary ATTR amyloidosis should have a known amyloidogenic TTR mutation demonstrated by genotyping AND is recognized to be primarily associated with CM AND one of the following:
    - Definite histochemical identification of amyloid by Congo red staining and green birefringence in crossed polarized light in cardiac or other tissue biopsy and identification of TTR as the amyloid fibril protein either by immunohistochemistry (IHC) or proteomic analysis.

**OR**

- - - Scintigraphy: ^99m^Tc-DPD with Grade 2 cardiac uptake or ^99m^Tc-PYP with either Grade 2 or 3 cardiac uptake.
  - Subjects with a diagnosis of wild-type ATTR-CM must be negative by genotyping AND have one of the following:
    - Definite histochemical identification of amyloid by Congo red staining and green birefringence in crossed polarized light in cardiac or other tissue biopsy and identification of TTR as the amyloid fibril protein either by IHC or proteomic analysis.

OR

- - - Scintigraphy ^99m^Tc-DPD with Grade 2 cardiac uptake or ^99m^Tc-PYP with Grade 2 or 3 cardiac uptake.
- Clinically stable in New York Heart Association (NYHA) class II or III for the 3 months preceding Screening.
- Left ventricle mass (LVM) on CMR >200 g.

#### Inclusion criteria for Group 2

Type of subject and diagnosis including disease severity:

- Subject medically diagnosed with light-chain (AL) amyloidosis that has required chemotherapy or an autologous stem cell transplant based upon AL amyloidosis confirmed by biopsy with IHC staining or proteomic identification of AL amyloid fibril type, in subjects with definite monoclonal gammopathy in whom causative mutations of amyloidogenic genes have been excluded.
- Clinically stable in NYHA class II or III for the 3 months preceding Screening.
- ≥6 months after completing any line of chemotherapy, or after autologous stem cell transplantation, and having attained either a very good partial response or a complete response, and without the need for hematological maintenance therapies.
- LVM on CMR >150 g.

#### Inclusion criteria for Group 3

Type of subject and diagnosis including disease severity:

- Newly diagnosed AL amyloidosis confirmed by:
  - Biopsy with IHC staining or proteomic identification of AL amyloid fibril type in subjects with definite monoclonal gammopathy in whom causative mutations of amyloidogenic genes have been excluded.
- Mayo stage II or IIIa.
- Confirmed free light chain complete response during the first three cycles of first-line chemotherapy where at least the first cycle has been with CyBorD.
- LVM on CMR >150 g.

### Exclusion criteria

Subjects were not eligible for inclusion in this study if any of the following criteria applied:

Concurrent conditions/medical history (includes liver function and QTc interval).

- CM primarily caused by non-amyloid diseases (eg, ischemic heart disease; valvular heart disease).
- Interval from the Q wave on the electrocardiogram (ECG) to point T using Fredericia's formula (QTcF) >500 msec.
- Sustained/symptomatic monomorphic ventricular tachycardia (VT), or rapid polymorphic VT, at Screening.
- Unstable heart failure defined as emergency hospitalization for worsening, or decompensated heart failure, or syncopal episode within 1 month of screening.
- Implantable cardiac defibrillator (ICD) or permanent pacemaker (PPM) at Screening
- NT-proBNP >8500 ng/L.
- Glomerular filtration rate (GFR) at Screening < 40 mL/min.
- Any active and persistent dermatological condition.
- Existing diagnosis of any type of dementia.
- History of allogeneic stem cell transplantation, prior solid organ transplant, or anticipated to undergo solid organ transplantation, or left ventricular assist device (LVAD) implantation, during the course of the study.
- Malignancy within last 5 years, except for basal or squamous cell carcinoma of the skin, or carcinoma in situ of the cervix that has been successfully treated. *Note: Subjects with a history of other malignancies that have been curatively treated may be eligible, but must be discussed with and approved by the Medical Monitor.* Previous or current diagnosis of symptomatic multiple myeloma.
- Acute coronary syndrome, or any form of coronary revascularization procedure (including coronary artery bypass grafting [CABG]), within 6 months of screening.
- Stroke within 6 months of screening, or transient ischemic attack within 3 months of screening.
- Symptomatic, clinically significant autonomic neuropathy which the Principal Investigator feels will preclude administration of study treatment.
- Hypoalbuminemia (serum albumin < 30 g/L).
- Uncontrolled hypertension during Screening.
- Alanine transaminase (ALT) >3x upper limit of normal (ULN) AND bilirubin >1.5xULN (isolated bilirubin >1.5xULN is acceptable if bilirubin is fractionated and direct bilirubin <35%).
- Peripheral edema at Screening that in the opinion of the Principal Investigator or designee might prevent adequate absorption of subcutaneously administered miridesap.
- Urine dipstick positive (>1+) for blood during Screening with investigation indicating glomerular hematuria. If other causes are identified, subjects may be enrolled on resolution of the abnormality.
- Presence of any comorbid or an uncontrolled medical condition (eg, diabetes mellitus), which in the opinion of the Investigator would increase the potential risk to the subject. Investigator should liaise with the Medical Monitor where there is uncertainty as to the eligibility of a patient.
- Positive test for hepatitis B hepatitis C, and/or human immunodeficiency virus (HIV) during Screening, or within 3 months prior to first dose of study treatment.

Concomitant medications

- Use of miridesap, or participation in a separate clinical trial involving miridesap within
  3 months of screening.
- Use of any of the following prohibited concomitant medication during the associated pre-Screen restriction period:
  - Tafamidis, diflunisal, doxycyline, tauroursodeoxycholic acid (TUDCA) (28 days)
  - Green tea extract (28 days)
  - Immunomodulatory drug (12 weeks)
  - Disease-modifying drug for any type of autoimmune disease (eg, rheumatoid arthritis), including but not restricted to, methotrexate, cyclophosphamide, or anti-cytokine antibodies (12 weeks)
  - Antibody therapy for amyloidosis treatment (at any time)
  - Conditioning chemotherapy for stem cell harvesting (6 weeks).

Diagnostic assessments and other criteria

- Donation of blood or blood products in excess of 500 mL within 84 days of Screening.
- Lactating females.
- Poor or unsuitable venous access.

Other

- Treatment with another investigational drug, biological agent, or device within 6 months of screening, or 5 half-lives of the study agent, whichever is longer.
- Unwillingness or inability to follow the procedures outlined in the protocol.

Contraindications

- History of sensitivity to any of the study medications, or metabolite thereof or a history of drug or other allergy that, in the opinion of the Investigator or Medical Monitor, contraindicates their participation.

CMR scanning

- Orthopnea of sufficient severity to preclude supine scanning as determined at Screening.
- Contraindication to magnetic resonance imaging (MRI) contrast agents.
- Inability to fit inside scanner due to body size (girth).
- Contraindication for MRI scanning (as assessed by local MRI safety questionnaire), which includes but is not limited to:
  - Intracranial aneurysm clips (except Sugita) or other metallic objects
  - Intra- orbital metal fragments that have not been removed
  - Pacemakers or other implanted cardiac rhythm management/monitoring devices and non-magnetic resonance (non-MR) conditional heart valves
  - Inner ear implants
  - History of claustrophobia.

^99m^TC-PYP **OR** ^99m^TC-DPD bone tracer radioscintography

- Orthopnea of sufficient severity to preclude supine scanning as determined at Screening.
- Previous allergic reaction to radioisotope bone tracers.
- Previous inclusion in a research protocol involving nuclear medicine, positron emission tomography (PET) or radiological investigations with significant radiation burden (a significant radiation burden being defined as 10 mSv in addition to natural background radiation, in the previous 3 years).

#### Exclusion criteria for Group 1

- Has any of the following:
  - Fulfillment of diagnostic criteria for AL amyloidosis
  - TTR polyneuropathy and/or intracranial TTR involvement including ophthalmological disease.
- Non-amyloidosis related chronic liver disease (with the exception of Gilbert’s syndrome or clinically asymptomatic gallstones*). Note: Stable chronic liver disease should generally be defined by the absence of ascites, encephalopathy, coagulopathy, hypoalbuminemia, esophageal or gastric varices, or persistent jaundice.*
- Platelet count <125 x 10^9^/L.

#### Exclusion criteria for Group 2

- Chronic liver disease or current active liver or biliary disease not attributable to amyloidosis (with the exception of Gilbert’s syndrome or asymptomatic gallstones). *Note: Stable chronic liver disease should generally be defined by the absence of ascites, encephalopathy coagulopathy, hypoalbuminemia, esophageal or gastric varices, or persistent jaundice.*

#### Exclusion criteria for Group 3

- Chronic liver disease or current active liver or biliary disease not attributable to amyloidosis (with the exception of Gilbert’s syndrome or asymptomatic gallstones). *Note: Stable chronic liver disease should generally be defined by the absence of ascites, encephalopathy, coagulopathy, hypoalbuminemia, esophageal or gastric varices, or persistent jaundice*
- Platelet count <75 x 10^9^/L

## Immuno-PET study

### Inclusion criteria

Age

- Subject must be 65 to 80 years of age inclusive, at the time of signing the informed consent.

Type of subject and disease characteristics

- Subjects with a diagnosis of ATTR-CM.
  - Wild-type ATTR status must be confirmed by genotyping AND have one of the following:
    - Definite histochemical identification of amyloid by Congo red staining and green birefringence in crossed polarized light in cardiac or other tissue biopsy and identification of TTR as the amyloid fibril protein either by immunohistochemistry or proteomic analysis

**OR**

- - - Scintigraphy Technetium-^99m^-labelled 3,3-diphosphono-1,2-propanodicarboxylic acid (^99m^Tc-DPD) with confirmed myocardial uptake
  - Hereditary ATTR amyloidosis (eg, TTR Val30Met) should have a known amyloidogenic TTR mutation demonstrated by genotyping AND is recognized to be primarily associated with CM AND one of the following:
    - Definite histochemical identification of amyloid by Congo red staining and green birefringence in crossed polarized light in cardiac or other tissue biopsy and identification of TTR as the amyloid fibril protein either by IHC or proteomic analysis. Note: Subjects with a confirmed mutation but who have not been biopsied may be eligible if they have an affected close blood relative whose amyloid has been confirmed histochemically. Such cases should be discussed with the Medical Monitor

**OR**

- - - Scintigraphy: ^99m^Tc-DPD with confirmed myocardial uptake.

Sex

- Both male and female subjects are eligible to participate.
  - Male subjects:
    - A male subject must agree to use one of the following methods of contraception during the treatment period and for at least 3 months after the last scan and refrain from donating sperm during this period:
      - Abstinence from penile-vaginal intercourse as their usual and preferred lifestyle (abstinent on a long-term and persistent basis) and agree to remain abstinent
      - Agree to use a male condom plus an additional method of contraception with a failure rate of <1% per year as described below when having penile-vaginal intercourse with a woman of childbearing potential
        - Combined (estrogen- and progestogen-containing) hormonal contraception associated with inhibition of ovulation

oral

intravaginal

transdermal

- - - - - Progestogen-only hormonal contraception associated with inhibition of ovulation

Injectable

- - - - - Implantable progestogen-only hormonal contraception associated with inhibition of ovulation

Intrauterine device

Intrauterine hormone-releasing system

bilateral tubal occlusion

- - - - - Vasectomy
        - Sexual abstinence
  - Female subjects:
    - A female subject is eligible to participate if she is not of childbearing potential, defined as:
      - Premenarchal
      - Premenopausal female with ONE of the following:
        - Documented hysterectomy
        - Documented bilateral salpingectomy
        - Documented bilateral oophorectomy

Note: Documentation can come from the site personnel’s: review of subject’s medical records, medical examination, or medical history interview

- - - - Postmenopausal female
        - A postmenopausal state is defined as no menses for 12 months without an alternative medical cause. A high FSH level in the postmenopausal range may be used to confirm a postmenopausal state in women not using hormonal contraception or hormonal HRT. However, in the absence of 12 months of amenorrhea, a single FSH measurement is insufficient.
      - Females on HRT and whose menopausal status is in doubt will be required to use one of the non-hormonal highly effective contraception methods if they wish to continue their HRT during the study. Otherwise, they must discontinue HRT to allow confirmation of postmenopausal status before study enrollment.

Other

- NYHA up to class III; subjects should be clinically stable for at least 3 months preceding to Screening.

### Exclusion criteria

Medical conditions

- CM primarily caused by non-amyloid diseases (eg, ischemic heart disease; valvular heart disease).
- Interval from the Q wave on the ECG to point T using Fredericia's formula (QTcF) >500 msec.
- Sustained (at a rate of ≥120 beats per min for ≥30 seconds), or symptomatic monomorphic VT, or rapid polymorphic VT, at Screening/Baseline cardiac monitoring.
- Systolic blood pressure ≤100 mmHg based on triplicate readings at screening.
- Unstable heart failure defined as emergency hospitalization for worsening, or decompensated heart failure, or syncopal episode within 1 month of screening.
- ICD or PPM at Screening.
- Estimated GFR at Screening <50 mL/min calculated using modification of diet in renal disease.
- Any active and persistent dermatological condition, which in the opinion of the Investigator and Medical Monitor would preclude safe participation.
- History of allogeneic stem cell transplantation, prior solid organ transplant, or anticipated to undergo solid organ transplantation, or LVAD implantation.
- Malignancy within last 5 years, except for basal or squamous cell carcinoma of the skin, or carcinoma in situ of the cervix that has been successfully treated. *Note: Subjects with a history of other malignancies that have been curatively treated may be eligible, but must be discussed with and approved by the Medical Monitor.*
- Acute coronary syndrome, or any form of coronary revascularization procedure (including CABG), within 6 months of screening.
- Symptomatic, clinically significant autonomic neuropathy which the Principal Investigator feels will preclude administration of study treatment.
- Uncontrolled hypertension during Screening.
- ALT >3x ULN OR bilirubin >1.5xULN (isolated bilirubin >1.5xULN is acceptable if bilirubin is fractionated and direct bilirubin <35%).
- Peripheral edema at Screening that in the opinion of the Principal Investigator or designee might prevent adequate absorption of subcutaneously administered miridesap.
- Presence of any comorbid (eg, steroid refractory rheumatoid arthritis), or an uncontrolled medical condition (eg, diabetes mellitus), which in the opinion of the Investigator would increase the potential risk to the subject. Investigator should liaise with the Medical Monitor where there is uncertainty as to the eligibility of a patient.
- Positive test for hepatitis B, hepatitis C, and/or HIV during Screening, or within 3 months prior to first dose of study treatment.
- Clinically significant multiple or severe drug allergies, intolerance to topical corticosteroids, or severe post-treatment hypersensitivity reactions (including, but not limited to, erythema multiforme major, linear immunoglobulin A dermatosis, toxic epidermal necrolysis, and exfoliative dermatitis).
- Inability to comprehend and/or understand the study patient information sheet, and/or unwillingness or inability to follow the procedures outlined in the protocol.
  ATTR-CM specific criteria
- Has any of the following:
  - Fulfillment of diagnostic criteria for AL amyloidosis
  - Fulfillment of diagnostic criteria for amyloid A or non-TTR hereditary amyloidosis.
- ATTR disease load:
  - Histologically proven or clinically sued gastrointestinal TTR amyloidosis
  - Diffuse skeletal muscle uptake of 99m(Tc)-DPD on Single-photon emission computed tomography imaging (where available)
  - Peripheral neuropathy causing more than mild morbidity (eg, walking disability; neuropathic pain affecting activities of daily living)
  - Proven or clinically suspected intracranial TTR involvement including ophthalmological disease.
- Non-amyloidosis related chronic liver disease (with the exception of Gilbert’s syndrome or clinically asymptomatic gallstones). *Note: Stable chronic liver disease should generally be defined by the absence of ascites, encephalopathy, coagulopathy, hypoalbuminemia, esophageal or gastric varices, or persistent jaundice.*

Prior/concomitant therapy

- Participation in a separate clinical trial involving miridesap within 3 months of screening
- Use of any of the following prohibited concomitant medication during the associated pre-Screen restriction period:
  - Tafamidis, diflunisal, TUDCA (initiation or change of dose within 28 days)
  - Green tea extract (initiation or change of dose within 28 days)
  - Silencing therapies (eg, small interfering ribonucleic acid [siRNA]) for ATTR protein synthesis (3 months)
  - Disease-modifying drug for any type of autoimmune disease, including but not restricted to, methotrexate, cyclophosphamide, or anti-cytokine antibodies (eg, anti-tumor necrosis factor-α monoclonal antibody [mAb]) (at any time)
  - Antibody therapy for amyloidosis treatment (at any time).

Prior/concurrent clinical study experience

- Treatment with another investigational drug, biological agent, or device within 6 months of screening, or 5 half-lives of the study agent, whichever is longer.

Diagnostic assessments for PET and CMR scanning

- Orthopnea of sufficient severity to preclude supine scanning as determined at Screening.
- Inability to fit inside scanner due to body size (girth).
- History of claustrophobia.
- Contraindication to MRI contrast agents.
- Contraindication for MRI scanning (as assessed by local MRI safety questionnaire), which includes but is not limited to:
  - Intracranial aneurysm clips (except Sugita) or other metallic objects
  - Intra-orbital metal fragments that have not been removed
  - Pacemakers or other implanted cardiac rhythm management/monitoring devices and non-MR conditional heart valves
  - Inner ear implants.

Other exclusions

- Donation of blood or blood products more than 500 mL within 84 days of screening.
- Poor or unsuitable venous access.
