# Supplementary material for: Pharmacodynamic evaluation and safety assessment of treatment with antibodies to serum amyloid P component in patients with cardiac amyloidosis: an open-label Phase 2 study and an adjunctive immuno-PET imaging study
Source: BMC Cardiovasc Disord. 2022 Feb 13;22:49. doi: 10.1186/s12872-021-02407-6 (PMC8843022; doi:10.1186/s12872-021-02407-6)
Supplement: Supplementary file 3 — Additional file 3. Study objectives. [file 12872_2021_2407_MOESM3_ESM.docx]

# Additional file 3

# Study objectives

A full, detailed list of all pre-defined primary, secondary and exploratory objectives can be found in the protocol.

## Phase 2 study

### Secondary objectives

- Investigation of rash associated with dezamizumab treatment via histopathological and IHC examination of skin biopsies and blood biomarkers (as data permitted).
- Characterization of the pharmacokinetics (PK) of anti-SAP mAb via descriptive PK parameters including the maximum concentration, the time associated with the maximum concentration, and the area under the concentration-time profile.
- Assessment of changes in circulating markers associated with pharmacodynamic effect (including but not limited to classical complement pathway components, acute phase proteins [eg, C-reactive protein, serum amyloid A protein and cytokines] during repeated administrations.
- Evaluation of changes in imaging markers of cardiac dysfunction (including, but not limited to strain [eg, global longitudinal strain], LV twist, stroke volume, ejection fraction, end diastolic volume and the ratio between early mitral inflow velocity and mitral annular early diastolic velocity]) monitored by serial CMR and/or echocardiogram (ECHO) over time from Baseline to 8-week follow-up

### Exploratory endpoints

- Change in cardiac extracellular volume (ECV) over time from baseline to 8-week follow-up
- Change in individual patient quality of life over time from Baseline to 8-week follow up
- Assessment of clinical cardiac functional improvement (change in 6-minute walk test distance from Baseline to 8-week follow-up; change in N-terminal pro-B-type natriuretic peptide
  (NT-proBMP) from Baseline to 8-week follow-up)
- Assessment of reduction in cardiac uptake of radioisotope bone tracers (^99m^Technetium-dicarboxypropane diphosphonate (^99m^Tc-DPD) or ^99m^Tc-pyrophosphate (^99m^Tc-PYP) uptake from Baseline to 8-week follow-up (Group 1 only)
- Change in overall body load and in affected organs (excluding cardiac load) on SAP scan assessment from Baseline to 8-week follow-up
- Assess the correlation between circulating biomarkers (NT-proBNP, Troponin T, cytokines) and structural and functional CMR measures over time
- Evaluate changes in imaging markers of cardiac structure (including LV wall thickness and LV mass) as monitored by serial CMR and/or ECHO imaging over time from Baseline to 8-week follow-up.
- Evaluate changes in imaging markers of cardiac tissue characterization (including late-gadolinium enhancement, native T1 and ECV) as monitored by serial CMR over time from Baseline to 8-week follow-up.
- Assess the correlation between myocardial perfusion using CMR and cardiac structural, functional, and tissue characterization as measured by CMR and/or ECHO.
- Assessment of the immunogenicity of dezamizumab when co-administered with miridesap by measurement of anti-drug antibodies before and after treatment with dezamizumab (Baseline and 8-week follow-up).
- To characterize the subject experience of dezamizumab treatment regimen via patient interviews completed over the telephone after the 8-week follow-up or Early Withdrawal Visit.

## Immuno-PET study

### Additional exploratory endpoints

- Visual comparison and scoring of regional PET signals or CMR signals over the myocardium.
- Regional derived PET and CMR parameters correlated within and across ATTR-CM patients.
- Number of “virtual visits” and number of additional remote assessments collected.
