# Supplementary material for: Pharmacodynamic evaluation and safety assessment of treatment with antibodies to serum amyloid P component in patients with cardiac amyloidosis: an open-label Phase 2 study and an adjunctive immuno-PET imaging study
Source: BMC Cardiovasc Disord. 2022 Feb 13;22:49. doi: 10.1186/s12872-021-02407-6 (PMC8843022; doi:10.1186/s12872-021-02407-6)
Supplement: Supplementary file 4 — Additional file 4. Phase 2 CMR methodology. [file 12872_2021_2407_MOESM4_ESM.docx]

# Additional file 4

# Phase 2 study CMR methodology

Each CMR imaging session was planned to take approximately 45–60 minutes, with a maximum scan time inside of the scanner of 90 minutes. Contrast-enhanced CMR scans using an intravenous injection of a gadolinium-based contrast agent (GBCA; gadoteric acid, gadoteridol, or gadobutrol) and non-contrast-enhanced CMR scans were performed at specified time points. A GBCA dose ≤0.1 mmol/kg was used at each contrast-enhanced scanning session.

Whenever possible, the contrast-enhanced CMR scan at Screening was performed after a subject has passed other eligibility requirements and served as the Baseline examination. For each patient, follow-up CMR examinations were performed on the same scanner as the Baseline examination. All CMR scans were reviewed at the site for clinical abnormalities. Image analysis for the CMR endpoints were performed by a central core lab.
