# Supplementary material for: Pharmacodynamic evaluation and safety assessment of treatment with antibodies to serum amyloid P component in patients with cardiac amyloidosis: an open-label Phase 2 study and an adjunctive immuno-PET imaging study
Source: BMC Cardiovasc Disord. 2022 Feb 13;22:49. doi: 10.1186/s12872-021-02407-6 (PMC8843022; doi:10.1186/s12872-021-02407-6)
Supplement: Supplementary file 5 — Additional file 5. Phase 1 immuno-PET study methods. [file 12872_2021_2407_MOESM5_ESM.docx]

# Additional file 5

**Phase 1 immuno-PET study methods**

**Study design and treatment**

This was an open-label, non-randomized, single-center, two-part [89Zr]Zr-dezamizumab PET imaging study (NCT03417830) including clinically stable patients with cardiac dysfunction caused by ATTR-CM (Figure 1B). It was conducted concurrently with the Phase 2 study. A screening visit and baseline assessments were conducted within 35 days of the first anti-SAP dosing session. For each treatment cycle, patients were admitted as inpatients for 2 weeks. SAP depletion was performed as described above for the Phase 2 study (the first day of SAP depletion was denoted Day 1). Dezamizumab was administered on Day 3 via IV infusion of non-radiolabeled dezamizumab plus a separate, concurrent IV infusion of [^89^Zr]Zr-dezamizumab formulated to give an injection dose of 37 MBq in 10 mg [^89^Zr]Zr-dezamizumab to a fixed total mass dose of 80 mg (first session for both patients) and fixed total mass dose of 500 mg (second session for Patient 1). This was followed by up to three serial PET scans; the timing of the scans was flexible and varied between patients.

The protocol was originally planned to have two parts: Part A, in which 3 patients were to receive up to two treatment sessions, each ~26 days in duration with a minimum of 1 month between the start of each dezamizumab infusion. In Part A, data were to be reviewed as they were being collected (i.e., “in-stream”) owing to the adaptive nature of the protocol. A formal interim analysis was planned to be conducted before progression to Part B, in which up to three patients were to receive one treatment session of ~26 days in duration. The study was terminated prior to completion of Part A, and as a result, neither the formal interim analysis nor Part B were conducted.

# Preparation and quality control of [^89^Zr]Zr-dezamizumab

[89Zr]Zr-dezamizumab was produced as described by Vosjan et al. (1). 89Zr was purchased from Perkin-Elmer (Boston, USA). [89Zr]Zr-dezamizumab was produced in compliance with current Good Manufacturing Practice at the Amsterdam UMC, VU University, Amsterdam, Netherlands. The procedures for radiolabeling dezamizumab with 89Zr were validated with respect to the final quality of the prepared conjugate. The product was stable during shipment from the manufacturing site (Amsterdam, Netherlands) to the administration site (Uppsala, Sweden).

In summary, dezamizumab 5 mg (100 mg/mL) was diluted with 910 µL 0.9% NaCl. The pH was adjusted to 8.9-9.1 with 0.1 M Na_2_CO_3_. This solution was added to 6.6 µL of 5 mM NCS-Bz-DFO in DMSO (Macrocyclics, Boston, Massachusetts, USA). The solution was shaken in a thermomixer for 30 minutes at 37˚C at 550 rpm. Next, the conjugated DFO-dezamizumab was purified by size exclusion chromatography (PD10, GE Healthcare) and the product collected in 20 mM Histidine + 200 mM sucrose pH 6.30±0.20.

Finally, DFO-dezamizumab was radiolabeled. To this end 200 µL 1M oxalic acid containing the required amount of ^89^Zr was mixed with 90 µL 2M Na_2_CO_3_ and reacted for 3 minutes. Next, 1 mL
0.5 M HEPES and 0.71 mL DFO-dezamizumab (~1.7 mg) were added and reacted for 60 minutes at room temperature while slowly shaken. After the incubation period [^89^Zr]-dezamizumab was purified by size exclusion chromatography using a PD10 column. The product was eluted in 20 mM histidine + 200 mM sucrose pH 6.30±0.20. The product was formulated to arrive at an injection dose of 37 MBq – 10 mg – 20 mL [^89^Zr]Zr-dezamizumab. The mean of the product pH was 6.28 ± 0.10.

The mean radiochemical purity as assessed by spin filter was 99.3 ± 0.1%. To this end, 4 µL of product was diluted with 96 µL eluent (5% DMSO and 95% 20 mM Histidine + 200 mM sucrose buffer) and applied on a microcon-30 centrifugal filter unit (Ultracel YM-30, regenerated cellulose, 30 kDa cut-off, Merck Millipore, Burlington, Massachusetts, USA). The solution was spun down for 7 minutes at 14000 rpm (Eppendorf 5430). The filter was washed twice with 100 µL eluent and spun down at 14000 rpm for 7 minutes after each wash step. The filtrate contained free ^89^Zr/^89^Zr-DFO, while the radiolabeled [^89^Zr]Zr-dezamizumab was left on the filter.

The mean radiochemical purity was 99.8 ± 0.2% and the mean protein integrity was 99.5 ± 0.1% as determined by size exclusion high-performance liquid chromatography using a superdex increase 200 10/30 GL size exclusion column (GE healthcare Life Sciences; USA) using a mixture of 0.05 M sodium phosphate, 0.15 M sodium chloride (pH 6.8) and 0.01 M NaN_3_ as the eluent at a flow rate of 0.75 mL/min. The mean immune reactive fraction as assessed by a serum amyloid P component (SAP) binding assay was 92.7 ± 0.4%. Sterility of each [^89^Zr]Zr-dezamizumab batch was assured by performing a media fill immediately after final filter sterilization of each batch. These procedures resulted in a sterile final product with endotoxin levels <0.2 EU/mL.

**Patient Population**

The PET study included males and females aged 65–80 years recruited in Sweden, with a diagnosis of ATTR-CM, NYHA class <III, and who were clinically stable for ≥3 months prior to Screening. The main exclusion criteria were CM caused by non-amyloid diseases, corrected QT interval >500 msec, sustained or symptomatic monomorphic, or rapid polymorphic ventricular tachycardia, systolic blood pressure ≤100 mm/Hg, unstable or decompensated heart failure. Full inclusion and exclusion criteria are provided in **Additional file 2.**

**Assessments**

PET imaging was carried out to assess radioactivity distribution in the body after administration of 37 MBq [^89^Zr]Zr-dezamizumab. The imaging session consisted of a 20-minute dynamic, gated acquisition of the thorax to include the entire heart, followed by a partial whole-body scan. Each PET session lasted for a total of ~60–90 minutes, and patients had up to three scans per dosing session within 5 days of [^89^Zr]Zr-dezamizumab administration. Multiple blood samples were collected for measurement of plasma concentrations of both radiolabeled and non-radiolabeled dezamizumab following the end of corresponding infusions. Safety was assessed by the monitoring of AEs, clinical laboratory tests, vital signs, ECGs, and physical examination.

**Statistical methodology**

The sample size for each study was based on recruitment feasibility for these patients with rare disease in a timely fashion (10 patients per planned group for the Phase 2 study, and up to a total of three patients each for Part A and Part B of the PET imaging trial). In addition, the calculation for the Phase 2 study took into consideration the level of precision in the estimated LVM change and the estimated rate of AEs. Assuming a standard deviation of 20 g for the change in LVM, the half-width for the 95% confidence interval (CI) of the estimated change in LVM at 8-week follow‑up based on the observed data was ±14 g. For both studies, no statistical analyses were performed but descriptive statistics were produced. In addition, for the Phase 2 study, planned Bayesian linear mixed models were not performed due to the early termination of the study.

For the immuno-PET study, the all-treated population comprised all patients who received ≥1 anti-SAP treatment including [^89^Zr]Zr-dezamizumab. The safety population comprised all patients who received ≥1 dose of miridesap, dezamizumab or [^89^Zr]Zr-dezamizumab. The PK population comprised patients from the all-treated population for whom a PK sample was obtained and analyzed.

For the Phase 2 study, the safety population comprised patients who received ≥1 dose of miridesap or dezamizumab and was used for the summary of all data including safety, efficacy, PD, and PK. Baseline refers to the latest assessment prior to first administration of anti-SAP treatment. The first administration of anti-SAP treatment is defined as the first administration of miridesap or dezamizumab for all endpoints.

**References**

1. Vosjan MJ, Perk LR, Visser GW, Budde M, Jurek P, Kiefer GE, et al. Conjugation and radiolabeling of monoclonal antibodies with zirconium-89 for PET imaging using the bifunctional chelate p-isothiocyanatobenzyl-desferrioxamine. Nat Protoc. 2010;5(4):739-43.
