# Supplementary material for: Pharmacodynamic evaluation and safety assessment of treatment with antibodies to serum amyloid P component in patients with cardiac amyloidosis: an open-label Phase 2 study and an adjunctive immuno-PET imaging study
Source: BMC Cardiovasc Disord. 2022 Feb 13;22:49. doi: 10.1186/s12872-021-02407-6 (PMC8843022; doi:10.1186/s12872-021-02407-6)
Supplement: Supplementary file 6 — Additional file 6. Median plasma dezamizumab concentration–time plots by treatment cycle (Phase 2 study; safety population). [file 12872_2021_2407_MOESM6_ESM.docx]

## Additional file 6

## Median plasma dezamizumab concentration-time plots by treatment cycle (Phase 2 study; safety population)


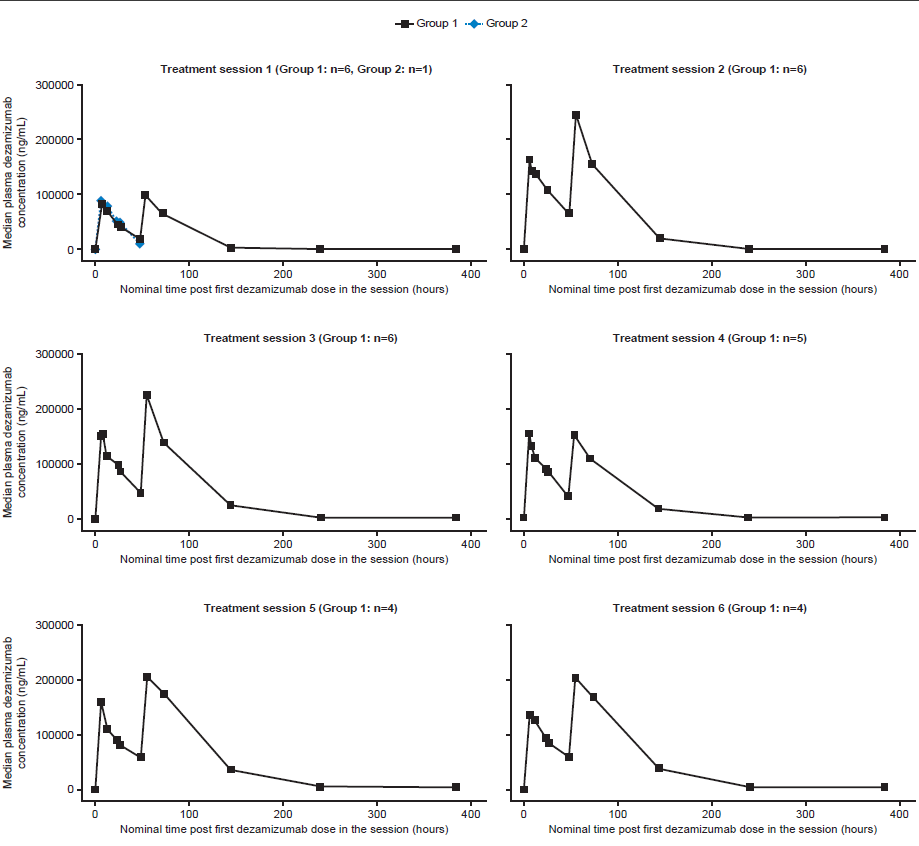


In Group 2, one patient received a partial dose of 300 mg dezamizumab on Day 1 of treatment session 1. No other patients were enrolled in Group 2.
