# Supplementary material for: Pharmacodynamic evaluation and safety assessment of treatment with antibodies to serum amyloid P component in patients with cardiac amyloidosis: an open-label Phase 2 study and an adjunctive immuno-PET imaging study
Source: BMC Cardiovasc Disord. 2022 Feb 13;22:49. doi: 10.1186/s12872-021-02407-6 (PMC8843022; doi:10.1186/s12872-021-02407-6)
Supplement: Supplementary file 7 — Additional file 7. Plasma PK of [89Zr]Zr-dezamizumab and total dezamizumab (non-radiolabelled dezamizumab and [89Zr]Zr-dezamizumab (immuno-PET study; PK population). [file 12872_2021_2407_MOESM7_ESM.docx]

## ADDITIONAL FILE 7

## Plasma PK of [^89^Zr]Zr-dezamizumab and total dezamizumab (non-radiolabeled dezamizumab and [^89^Zr]Zr-dezamizumab) (immuno-PET study; PK population)

| **PK parameter** | **Patient A** | | **Patient B** |
| --- | --- | --- | --- |
| **[^89^Zr]Zr-dezamizumab**  **dose (mg)** | **Session 1**  **9.94** | **Session 2**  **9.93** | **Session 1**  **10.38** |
| AUC(0-t) (h*Bq/g) | 244037 | 386077 | 92052 |
| AUC(0-inf) (h*Bq/g) | 253096 | 457666 | 96432 |
| C_max_ (Bq/g) | 11882 | 11923 | 6599 |
| T_max_ (h) | 1.15 | 1.1 | 1.17 |
| t_½_ (h) | 15.2 | 26.7 | 13.8 |
| **Total dezamizumab**  **dose (mg)** | 79.94 | 499.83 | 80.38 |
| AUC(0-t) (h*ng/mL) | 768518 | 8274231 | 173964 |
| AUC(0-inf) (h*ng/mL) | 788651 | 8696309 | 186190 |
| C_max_ (ng/mL) | 28648 | 226994 | 7474 |
| T_max_ (h) | 1.02 | 6.1 | 5 |
| t_½_ (h) | 13.5 | 23.1 | 21.9 |

0-inf, from time 0 to infinity; 0-t, from time 0 to last measurable concentration; AUC, area under curve; C_max_, maximum concentration; PET, positron emission tomography; PK, pharmacokinetics; t_½_, half-life; T_max_, time to reach C_max._
