# Supplementary material for: Pharmacodynamic evaluation and safety assessment of treatment with antibodies to serum amyloid P component in patients with cardiac amyloidosis: an open-label Phase 2 study and an adjunctive immuno-PET imaging study
Source: BMC Cardiovasc Disord. 2022 Feb 13;22:49. doi: 10.1186/s12872-021-02407-6 (PMC8843022; doi:10.1186/s12872-021-02407-6)
Supplement: Supplementary file 8 — Additional file 8. Changes in LVM across treatment sessions for each patient (Phase 2 study). [file 12872_2021_2407_MOESM8_ESM.docx]

# **Additional file 8**

## **Changes in LVM across treatment sessions for each patient (Phase 2 study)**

**Group 1**


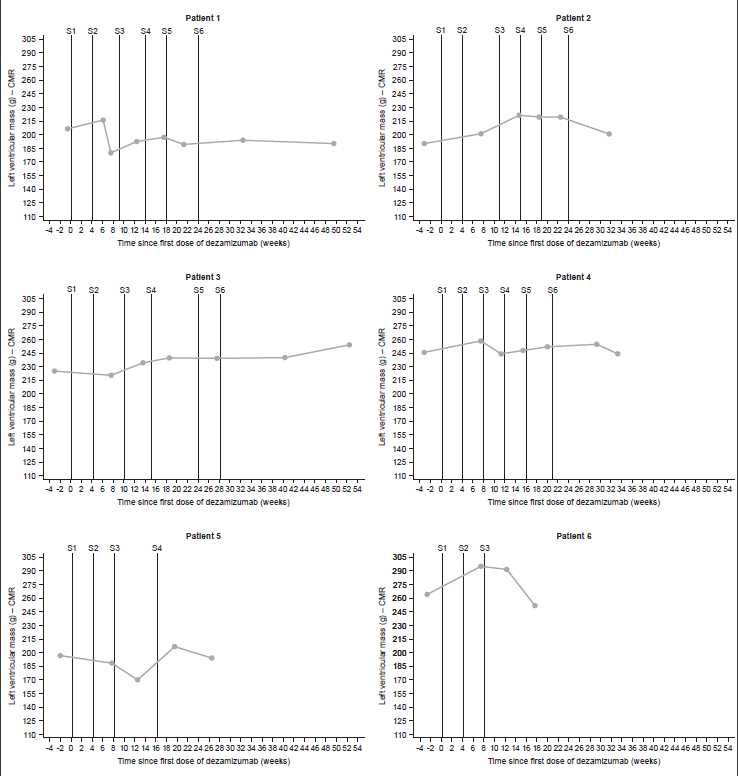


**Group 2**


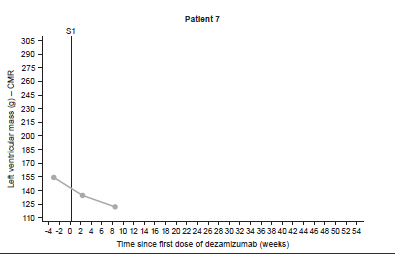


CMR, cardiac magnetic resonance; LVM, left ventricular mass.
