# Supplementary material for: Pharmacodynamic evaluation and safety assessment of treatment with antibodies to serum amyloid P component in patients with cardiac amyloidosis: an open-label Phase 2 study and an adjunctive immuno-PET imaging study
Source: BMC Cardiovasc Disord. 2022 Feb 13;22:49. doi: 10.1186/s12872-021-02407-6 (PMC8843022; doi:10.1186/s12872-021-02407-6)
Supplement: Supplementary file 9 — Additional file 9. Summary of imaging markers of cardiac dysfunction as measured by CMR and/or ECHO imaging over time (safety population).Summary of imaging markers of cardiac dysfunction as measured by CMR and/or ECHO imaging over time (safety population). [file 12872_2021_2407_MOESM9_ESM.docx]

## ADDITIONAL FILE 9

## Summary of imaging markers of cardiac dysfunction as measured by CMR and/or ECHO imaging over time (safety population)

|  | Visit (Baseline and change from Baseline)* |  |  | **Parameter Measure** | | |  |
| --- | --- | --- | --- | --- | --- | --- | --- |
| Dezamizumab  Treatment |  | n | Mean | SD | Median | Min. | Max. |
| Global Longitudinal Strain Tagging (%) - CMR | | | | | | | |
| Group 1 | Baseline | 6 | -5.638 | 1.0306 | -5.753 | -6.86 | -4.37 |
|  | 8-week follow-up | 6 | 1.125 | 1.6448 | 0.936 | -0.51 | 4.06 |
| Group 2 | Baseline | 1 | -3.497 |  | -3.497 | -3.50 | -3.50 |
|  | 8-week follow-up | 1 | -3.404 |  | -3.404 | -3.40 | -3.40 |
| Global Longitudinal Strain Feature Tracking (%) - CMR | | | | | | | |
| Group 1 | Baseline | 6 | -6.797 | 1.7298 | -7.097 | -8.88 | -4.81 |
|  | 8-week follow-up | 6 | 0.639 | 1.3678 | 0.707 | -1.34 | 2.46 |
| Group 2 | Baseline | 1 | -9.754 |  | -9.754 | -9.75 | -9.75 |
|  | 8-week follow-up | 1 | 3.951 |  | 3.951 | 3.95 | 3.95 |
| Global Longitudinal Strain Speckle Tracking (%) - ECHO | | | | | | | |
| Group 1 | Baseline | 6 | -18.22 | 2.971 | -17.90 | -22.8 | -14.8 |
|  | 8-week follow-up | 6 | -1.82 | 10.013 | -1.25 | -13.2 | 12.1 |
| Group 2 | Baseline | 1 | -30.30 |  | -30.30 | -30.3 | -30.3 |
|  | 8-week follow-up | 1 | 1.60 |  | 1.60 | 1.6 | 1.6 |
| Twist (degrees) - CMR | | | | | | | |
| Group 1 | Baseline | 6 | 4.331 | 1.6119 | 4.160 | 1.85 | 6.76 |
|  | 8-week follow-up | 6 | -0.264 | 2.0835 | -0.507 | -2.87 | 3.50 |
| Group 2 | Baseline | 1 | 6.957 |  | 6.957 | 6.96 | 6.96 |
|  | 8-week follow-up | 1 | 0.914 |  | 0.914 | 0.91 | 0.91 |
| Left Ventricular Stroke Volume (mL) - CMR | | | | | | | |
| Group 1 | Baseline | 6 | 77.982 | 16.8373 | 71.640 | 63.45 | 105.66 |
|  | 8-week follow-up | 6 | 2.400 | 19.1252 | 3.715 | -18.01 | 21.35 |
| Group 2 | Baseline | 1 | 92.680 |  | 92.680 | 92.68 | 92.68 |
|  | 8-week follow-up | 1 | -6.750 |  | -6.750 | -6.75 | -6.75 |
| Left Ventricular Stroke Volume (mL) - ECHO | | | | | | | |
| Group 1 | Baseline | 6 | 42.530 | 6.7163 | 40.980 | 36.04 | 53.42 |
|  | 8-week follow-up | 6 | -4.343 | 7.3457 | -5.895 | -10.19 | 9.85 |
| Group 2 | Baseline | 1 | 41.130 |  | 41.130 | 41.13 | 41.13 |
|  | 8-week follow-up | 1 | -6.490 |  | -6.490 | -6.49 | -6.49 |

|  | Visit (Baseline and change from Baseline)* |  |  | **Parameter Measure** | | |  |
| --- | --- | --- | --- | --- | --- | --- | --- |
| Dezamizumab  Treatment |  | n | Mean | SD | Median | Min. | Max. |
| Left Ventricular Ejection Fraction (%) - CMR | | | | | | | |
| Group 1 | Baseline | 6 | 50.837 | 8.8004 | 48.055 | 44.88 | 68.52 |
|  | 8-week follow-up | 6 | -1.258 | 7.9932 | -0.960 | -13.97 | 8.44 |
| Group 2 | Baseline | 1 | 76.050 |  | 76.050 | 76.05 | 76.05 |
|  | 8-week follow-up | 1 | -2.800 |  | -2.800 | -2.80 | -2.80 |
|  |  | Left Ventricular Ejection Fraction (%) - ECHO | | | | | |
| Group 1 | Baseline | 6 | 52.57 | 8.694 | 55.35 | 40.3 | 60.5 |
|  | 8-week follow-up | 6 | -1.72 | 8.471 | -5.95 | -8.4 | 11.4 |
| Group 2 | Baseline | 1 | 66.30 |  | 66.30 | 66.3 | 66.3 |
|  | 8-week follow-up | 1 | -3.20 |  | -3.20 | -3.2 | -3.2 |
|  |  | Left Ventricular End Diastolic Volume (mL) - CMR | | | | | |
| Group 1 | Baseline | 6 | 155.818 | 40.1535 | 141.745 | 130.72 | 235.42 |
|  | 8-week follow-up | 6 | 9.887 | 19.3925 | 14.165 | -15.71 | 33.42 |
| Group 2 | Baseline | 1 | 121.870 |  | 121.870 | 121.87 | 121.87 |
|  | 8-week follow-up | 1 | -4.560 |  | -4.560 | -4.56 | -4.56 |
|  |  | Left Ventricular End Diastolic Volume (mL) - ECHO | | | | | |
| Group 1 | Baseline | 6 | 82.982 | 20.1794 | 80.585 | 59.53 | 116.76 |
|  | 8-week follow-up | 6 | -7.648 | 4.4630 | -7.235 | -14.90 | -1.79 |
| Group 2 | Baseline | 1 | 62.050 |  | 62.050 | 62.05 | 62.05 |
|  | 8-week follow-up | 1 | -7.100 |  | -7.100 | -7.10 | -7.10 |
|  |  | E/e’ Lateral Ratio - ECHO | | | | | |
| Group 1 | Baseline | 6 | 14.25 | 7.217 | 13.55 | 6.1 | 27.0 |
|  | 8-week follow-up | 6 | 0.05 | 2.681 | 0.25 | -4.3 | 2.8 |
| Group 2 | Baseline | 1 | 16.20 |  | 16.20 | 16.2 | 16.2 |
|  | 8-week follow-up | 1 | 2.90 |  | 2.90 | 2.9 | 2.9 |
|  |  | E/e’ Septal Ratio - ECHO | | | | | |
| Group 1 | Baseline | 6 | 20.10 | 8.047 | 18.25 | 13.7 | 34.9 |
|  | 8-week follow-up | 6 | 2.83 | 4.236 | 2.65 | -2.7 | 9.2 |
| Group 2 | Baseline | 1 | 16.30 |  | 16.30 | 16.3 | 16.3 |

*Baseline was defined as the latest assessment prior to the first administration of either dezamizumab or miridesap.

CMR, cardiac magnetic resonance; ECHO, echocardiogram; E/e’, ratio between early mitral inflow velocity and mitral annular early diastolic velocity; LV, left ventricular; SD, standard deviation.
